# Oxidation Kinetics of Nanocrystalline Hexagonal RMn_1–*x*_Ti_*x*_O_3_ (R = Ho, Dy)

**DOI:** 10.1021/acsami.3c06020

**Published:** 2023-08-28

**Authors:** Frida Hemstad Danmo, Inger-Emma Nylund, Aamund Westermoen, Kenneth P. Marshall, Dragos Stoian, Tor Grande, Julia Glaum, Sverre M. Selbach

**Affiliations:** † Department of Materials Science and Engineering, NTNU Norwegian University of Science and Technology, NO-7491 Trondheim, Norway; ‡ The Swiss-Norwegian Beamlines (SNBL), European Synchrotron Radiation Facility, Grenoble 38043, France

**Keywords:** oxygen absorption, oxygen exchange, oxygen storage materials, oxidation kinetics, hexagonal manganites

## Abstract

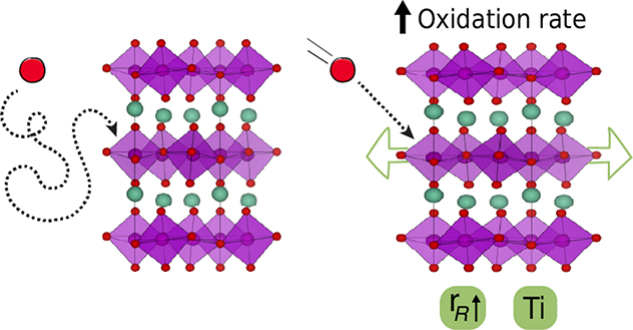

Hexagonal manganites, RMnO_3_ (R = Sc, Y, Ho-Lu), are potential oxygen storage materials for air separation due to their reversible oxygen storage and release properties. Their outstanding ability to absorb and release oxygen at relatively low temperatures of 250–400 °C holds promise of saving energy compared to current industrial methods. Unfortunately, the low temperature of operation also implies slow kinetics of oxygen exchange in these materials, which would make them inefficient in applications such as chemical looping air separation. Here, we show that the oxidation kinetics of RMnO_3_ can be improved through Ti^4+^-doping as well as by increasing the rare earth cation size. The rate of oxygen absorption of nanocrystalline RMn_1–*x*_Ti_*x*_O_3_ (R = Ho, Dy; *x* = 0, 0.15) was investigated by thermogravimetric analysis, X-ray absorption near-edge structure, and high-temperature X-ray diffraction (HT-XRD) with in situ switching of atmosphere from N_2_ to O_2_. The kinetics of oxidation increases for larger R and even more with Ti^4+^ donor doping, as both induce expansion of the *ab*-plane, which reduces the electrostatic repulsion between oxygen in the lattice upon oxygen ion migration. Surface exchange rates and activation energies of oxidation were determined from changes in lattice parameters observed through HT-XRD upon in situ switching of atmosphere.

## Introduction

Oxygen gas is imperative to a number of diverse applications, such as medical treatments, and production of steel, polymer materials, and pharmaceuticals.^1^ The demand for pure oxygen gas is already high and increasing every year. The emerging potential for using oxygen in oxy-combustion technology for CO_2_ capture may also further increase the demand in the coming years.^2^ Today, the bulk supply of oxygen is made through the highly energy-demanding process of cryogenic distillation.^3,4^ In recent years, air separation by oxygen absorption has been studied as a promising energy-efficient alternative for oxygen production.^5^ These methods are usually referred to as chemical looping air separation (CLAS) and utilize materials with reversible oxygen storage and release properties, known as “oxygen storage materials” (OSMs),^6^ in an either pressure swing- or temperature swing-based method. A suitable material for CLAS can rapidly absorb and release large amounts of oxygen, preferably at lower temperatures to reduce energy costs, and must not degrade over time.^2^

Possible oxygen carriers include transition metal oxides, ranging from binary manganese, iron, and copper oxides to complex ternary metal oxides.^7^ Perovskite-type oxides (ABO_3−δ_), such as La_*x*_Sr_1–*x*_MO_3_ (M = Mn, Co, Fe), CaMnO_3_, or SrFeO_3_-based materials, are possible OSMs due to their high oxygen storage capacities (OSC) and as their properties can be fine-tuned due to having broad cation compositional flexibility.^8−13^ These materials, which are widely studied transition metal-based oxygen carriers, utilize oxygen vacancies for the storage and transport of oxygen and allow for reversible switching between a reduced ABO_3−δ_ state and an oxidized ABO_3_ state depending on temperature and partial pressure of oxygen, *p*_O2_. These oxygen transport properties also make perovskite oxides promising material candidates within applications such as solid oxide fuel cells and metal–air batteries.^14,15^ Generally, bulk transport of oxygen is rate-limiting for particles larger than ∼1 μm, while the catalytic splitting or formation of the O_2_ molecule on the surface is rate-limiting for smaller particles.^16^ As oxygen vacancies in perovskites have limited mobility, temperatures as high as 600–800 °C are generally required to achieve sufficiently rapid oxygen exchange.^8,17^ At such elevated temperatures, these materials are prone to degradation and kinetic demixing.^18^ The OSC and oxygen exchange kinetics can be improved by doping on the A-site, B-site, or both.^10,19^ Compared to Sr_0.8_Ca_0.2_FeO_3_ and CaMnO_3_, Sr_0.8_Ca_0.2_Fe_0.4_Co_0.6_O_3−δ_ and Ca_0.8_Sr_0.2_MnO_3_ show faster surface oxygen exchange kinetics, shorter times required for oxidation, and smaller activation energy for bulk oxygen diffusion.^12,19^ Similar kinetics have also been found for SrFeO_3_-based materials^20,21^ making both material systems good candidates for CLAS at lower temperatures.

**Figure 1:**
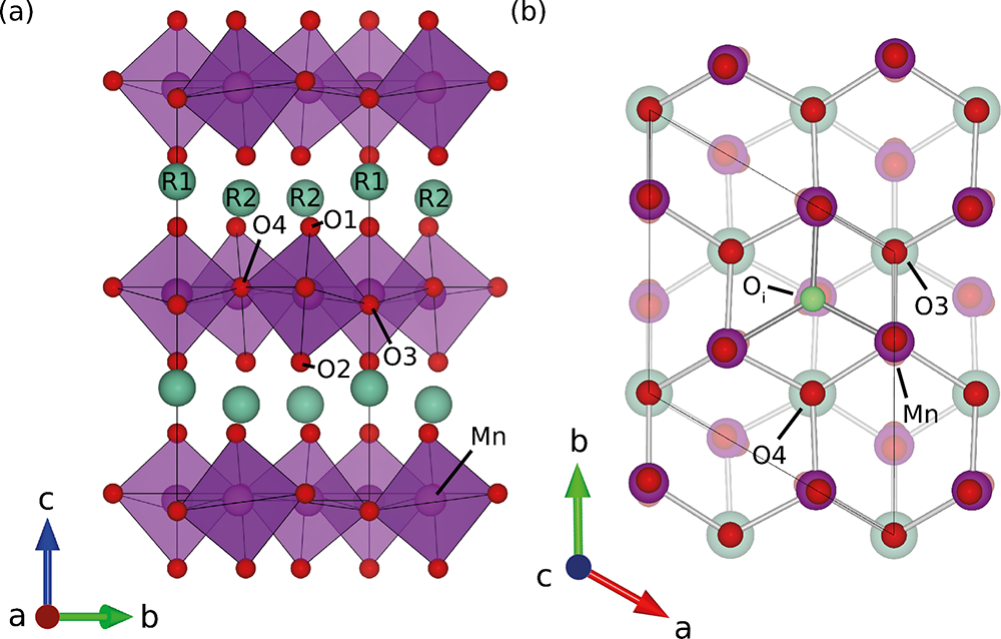
(a) Hexagonal *P*6_3_*cm* crystal structure with turquoise R^3+^ cations, red oxygen atoms, and the MnO_5_ trigonal bipyramids shown as purple polyhedra. (b) Mn–O plane is seen along the *c* axis, with the added O_i_ (green) on the interstitial site in between Mn. Figures are made using VESTA.^45^

Hexagonal manganites, RMnO_3+δ_ (R = Sc, Y, Ho-Lu), are possible OSMs due to their large OSCs at lower temperatures.^22−26^ RMnO_3_ oxides can crystallize in one of two crystal structures depending on the size of the R^3+^ cation, with smaller cations (*r*_R_^3+^ ≤ *r*_Ho_^3+^) favoring the layered hexagonal structure of interest here with space group *P*6_3_*cm* (Figure 1), while larger cations (*r*_R_^3+^ ≥ *r*_Dy_^3+^) favors the orthorhombic perovskite *Pnma* structure.^27^ The hexagonal *P*6_3_*cm* structure is less close-packed than the *Pnma* perovskite structure, and excess oxygen is accommodated as interstitials in the hexagonal polymorph, in contrast to cation vacancies in the perovskite structure.^28−30^ Calculated energy barriers for migration of interstitial oxygen through an interstitialcy mechanism are about 0.4–0.6 eV and lower compared to most oxygen vacancy migration barriers found in perovskites,^28,31−33^ enabling significant transport of oxygen interstitials at temperatures even below 200 °C. Unfortunately, at such low temperatures, these materials suffer from slow oxidation kinetics in bulk form. Even nanocrystalline samples, which generally show much faster oxidation kinetics than bulk materials,^34^ show reduced OSC with faster cooling rates.^25,35−40^ The maximum OSC of hexagonal manganites has been improved by R-site dopants.^35,37,38,41^ However, improving the kinetics of oxygen exchange is crucial for potential industrial applications. E.g. doping YMnO_3+δ_ with larger R cations results in oxygen contents as high as δ = 0.45 at slow cooling rates in oxygen atmosphere.^35^ Unfortunately, as these compositions have relatively slow redox kinetics, only a portion of the full capacity can be utilized within the timeframes needed for efficient temperature or pressure swing CLAS. Nanocrystalline Y_0.95_Pr_0.05_MnO_3_ has been shown to only need minutes to reduce from δ = 0.2 to δ = 0.0 in air at *T* > 270 °C, while reoxidation to δ = 0.2 requires several hours, as this occurs at a lower temperature where the redox kinetics are slower.^37^ Similar oxidation timeframes have also been reported for undoped YMnO_3_ and HoMnO_3_.^25,36^ Larger R cations do seem to affect the redox kinetics, as using R larger than Dy^3+^ has resulted in improved oxidation rates for R_0.25_Y_0.75_MnO_3_ (R = Tb, Gd, Sm) and Y_0.6_Tb_0.2_Ce_0.2_MnO_3_,^38,41^ and donor doping can increase both the OSC and the thermal stability of interstitial oxygen in hexagonal manganites.^40,42,43^ Ti^4+^ donor doping of the Mn sublattice^44^ has been reported to increase the absorption of oxygen and thermal stability of O_i_ in hexagonal manganites,^40,42^ and there are also indications that Ti^4+^-doping can improve the oxygen exchange kinetics.^40^ Ti^4+^ as a donor dopant is charge compensated by formal reduction of Mn^3+^ to Mn^2+^ at high temperatures or low *p*O_2_ where there is limited absorption of oxygen interstitials. At lower temperatures and higher *p*O_2_, Ti^4+^ is charge compensated by oxygen interstitials.^40^ The rate-limiting step for cyclic processes like CLAS is oxidation, and the oxidation kinetics of hexagonal manganites must be improved to enable commercial applications.

Here, we study the oxidation kinetics of RMn_1–*x*_Ti_*x*_O_3_ (R = Ho, Dy; *x* = 0, 0.15) by thermogravimetry and high-temperature X-ray diffraction (HT-XRD) and X-ray absorption spectroscopy with in situ switching of atmosphere. The oxidation kinetics are improved with the increasing size of R^3+^ and with 15% Ti^4+^ donor doping. Larger R^3+^ and particularly Ti^4+^ doping cause an expansion of the *ab*-plane and a lower ferroelectric Curie temperature, and both effects are suggested to improve the oxygen bulk transport. Surface exchange rates and activation energies were calculated from lattice parameter changes, using chemical expansion to gauge the oxygen content. The surface exchange kinetics are similar to literature reports on state-of-the-art materials measured at significantly higher temperatures. Finally, we discuss future avenues for further improving the oxidation kinetics of hexagonal manganites by aliovalent doping.

## Methods

### Synthesis

Nanocrystalline RMn_1–*x*_Ti_*x*_O_3_ samples were prepared using a previously reported modified citric acid synthesis route.^40,46^ Metal acetates Ho(CH_3_CO_2_)_3_·*x*H_2_O (AlfaAesar), Dy(CH_3_CO_2_)_3_·*x*H_2_O (AlfaAesar), and Mn(CH_3_CO_2_)_3_·*x*H_2_O (Riedel-de-Haën) were each dissolved in a mixture of citric acid (99% Sigma-Aldrich) and deionized water with a molar ratio of cation to citric acid of 1:20 for Ho^3+^, 1:35 for Dy^3+^, and 1:5 for Mn^3+^. The precursor solutions were stirred on a hot plate set to 150 °C until clear. For the Ti^4+^ precursor solution, titanium(IV) isopropoxide (TTIP) (Sigma-Aldrich) was added to citric acid dissolved in deionized water, with a molar ratio of cation to citric acid of 1:6.3, and stirred on a hot plate set to 60 °C until clear. The cation precursors were mixed in stoichiometric amounts, and ethylene glycol (EG) (Merck) was added with a 1:1 molar ratio between EG and citric acid. The solutions were stirred on a hot plate set to 150 °C until a viscous gel was formed, which was then dried at 120 °C for 3 days. The dried gels were heated to 400 °C and kept for 3 h and subsequently calcined at 600 °C for 6 h. The calcined amorphous powders were then crystallized at temperatures ranging from 850 to 950 °C in N_2_ atmosphere for 1 h. Some of the samples were pre-annealed in 5% H_2_ in N_2_ atmosphere at temperatures ranging from 250 to 300 °C prior to crystallization to achieve phase pure powders.^40^

### Characterization

To investigate phase purity and lattice parameters, XRD was performed using a Bruker D8 Focus with Cu Kα radiation. Lattice parameters and average crystallite size of the different powders (Table 1) were determined by Pawley refinement using Bruker AXS TOPAS 5.^47^ All samples were refined within the *P*6_3_*cm* space group. The DyMnO_3_ sample contains 14 molar% Dy_2_O_3_, inferred from Rietveld refinement. Oxygen stoichiometry as a function of temperature was analyzed by thermogravimetric analysis (TGA) using a Netzsch STA 449C Jupiter with 30 mL min^–1^ O_2_ gas flow. The samples were heated to and subsequently cooled from 800 °C using heating rates of 5, 10, and 20 °C min^–1^. Calculations of δ were based on a reference point chosen at the lowest mass at *T* > 700 °C where all Mn is assumed to be found as Mn^3+^, corresponding to δ = 0 and δ = 0.075 for undoped and Ti-doped samples, respectively. Prior to the TGA measurements, all samples were heated to 600 °C for 10 h in N_2_ atmosphere to remove excess oxygen.

**Table 1:** Composition, Naming, and Pawley-Refined Lattice Parameters and Crystallite Size of As-Synthesized RMn_1–*x*_Ti_*x*_O_3_ Samples^a^

composition	crystallite size (nm)	*a* (Å)	*c* (Å)	volume (Å^3^)	*c*/*a*	*R*_wp_ (%)
HoMnO_3_	25 ± 1	6.131(0)	11.371(0)	370.2	1.854	6.54
HoMn_0.85_Ti_0.15_O_3_	28 ± 1	6.141(8)	11.400(0)	372.4	1.856	8.51
DyMnO_3_	30 ± 1	6.162(3)	11.368(8)	373.9	1.844	11.12
DyMn_0.85_Ti_0.15_O_3_	30 ± 1	6.199(3)	11.433(3)	380.5	1.844	2.91

a All samples were refined within the *P*6_3_*cm* space group.

**Figure 2:**
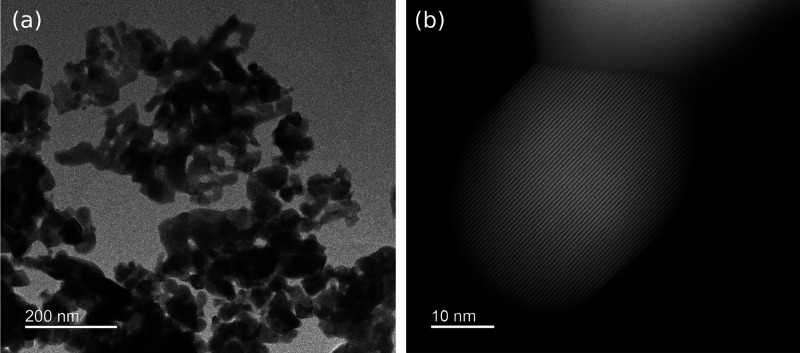
(a) BF TEM image of the nanocrystalline HoMnO_3_ powder. (b) STEM image of a crystallite in the HoMn_0.85_Ti_0.15_O_3_ sample.

The morphology of the samples was studied using transmission electron microscopy (TEM) imaging, performed on an aberration-corrected Jeol JEM ARM200F equipped with a cold field emission gun operated at 200 kV. Bright-field TEM and scanning TEM (STEM) images were acquired and are shown in Figure 2 for HoMnO_3_ and HoMn_0.85_Ti_0.15_O_3_. For the STEM image acquisition, a beam semi-convergence angle of 27 mrad and collection angles of 67–155 mrads were used.

HT-XRD and X-ray absorption near-edge structure (XANES) measurements with in situ switching of atmosphere were carried out at the BM31 beamline of the Swiss-Norwegian Beamlines (SNBL) at the European Synchrotron Radiation Facility (ESRF) in Grenoble, France. A few milligrams of each sample were each placed in a capillary with quartz wool on each side. All experiments were performed using 10 mL min^–1^ gas flow, which was verified using a Pfeiffer Vacuum Omnistar spectrometer. For diffraction measurements, a wavelength of 0.338591 Å and a Dexela 2923 area detector were used. A Hitachi Vortex single-element silicon drift detector was used to measure the XANES fluorescence signal. Absorption measurements were done in fluorescence mode, as the transmission for Mn was weak due to the heavy rare earth elements attenuating the signal. XANES measurements were performed prior to, and after, each XRD measurement. In addition, each sample was measured once using XANES with in situ switching of atmosphere at a temperature chosen for each sample to display reasonable kinetics. Prior to the synchrotron measurements, all samples were heated to 600 °C in N_2_ atmosphere to remove any excess oxygen from the structure. The samples were heated 500 °C in N_2_ atmosphere in between each measurement. TOPAS 5 operating in launch mode was used for Pawley refinement of lattice parameters. Larch^48^ was used for the normalization of XANES measurements.

## Results

**Figure 3:**
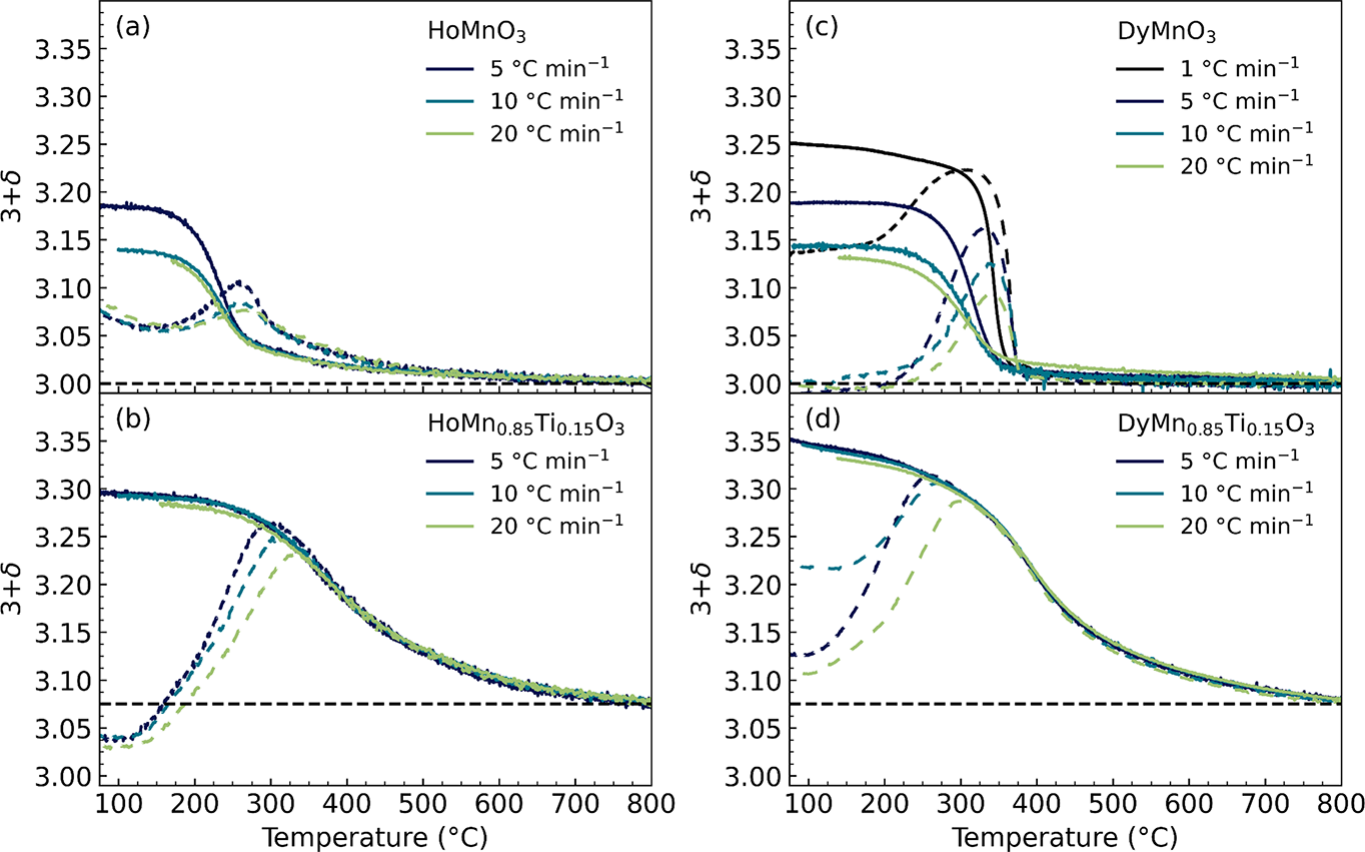
Oxygen stoichiometry 3 + δ measured by TGA during heating (dashed lines) and cooling (solid lines) in O_2_ atmosphere using different heating and cooling rates for (a) HoMnO_3_, (b) HoMn_0.85_Ti_0.15_O_3_, (c) DyMnO_3_, and (d) DyMn_0.85_Ti_0.15_O_3_. The horizontal dashed lines indicate the oxygen stoichiometry where all Mn is assumed to be Mn^3+^; for Ti-doped samples, this corresponds to δ = 0.075.

The changes in oxygen stoichiometry as a function of temperature, measured by TGA at different heating and cooling rates in O_2_ atmosphere, are shown in Figure 3. All samples show oxygen absorption at temperatures below ∼350 °C during heating and cooling, with the maximum oxygen stoichiometry during heating found at ∼300 °C. The maximum oxygen stoichiometry decreases with increasing heating rates as less time is spent in the temperature region where oxidation occurs. All samples reach a plateau in oxygen stoichiometry when cooling to temperatures below 200 °C, where further oxidation is hindered by slower kinetics at low temperatures. For the undoped samples in panels (a) and (c), the oxygen stoichiometry at this plateau decreases significantly with faster cooling rates. These materials also display a significant thermal hysteresis in oxygen stoichiometry during heating and cooling, and the width of the hysteresis for DyMnO_3_ increases from ∼30 to ∼60 °C when increasing the rate from 1 to 5 °C min^–1^. No differences in the kinetics of oxidation between HoMnO_3_ and DyMnO_3_ can be inferred from the TGA measurements.

In contrast to the undoped materials, the Ti-doped materials (Figure 3b,d) show no thermal hysteresis in oxygen stoichiometry upon heating and cooling, and the OSC is insensitive to increasing cooling rates. During heating, these samples start to oxidize at much lower temperatures than undoped samples, with temperature onsets of oxidation at ∼100 °C for HoMn_0.85_Ti_0.15_O_3_ and DyMn_0.85_Ti_0.15_O_3_, compared to ∼150 and ∼200 °C for HoMnO_3_ and DyMnO_3_, respectively. This indicates that Ti^4+^ donor doping greatly improves the kinetics of oxidation as well as that it stabilizes oxygen interstitials to higher temperatures.

**Figure 4:**
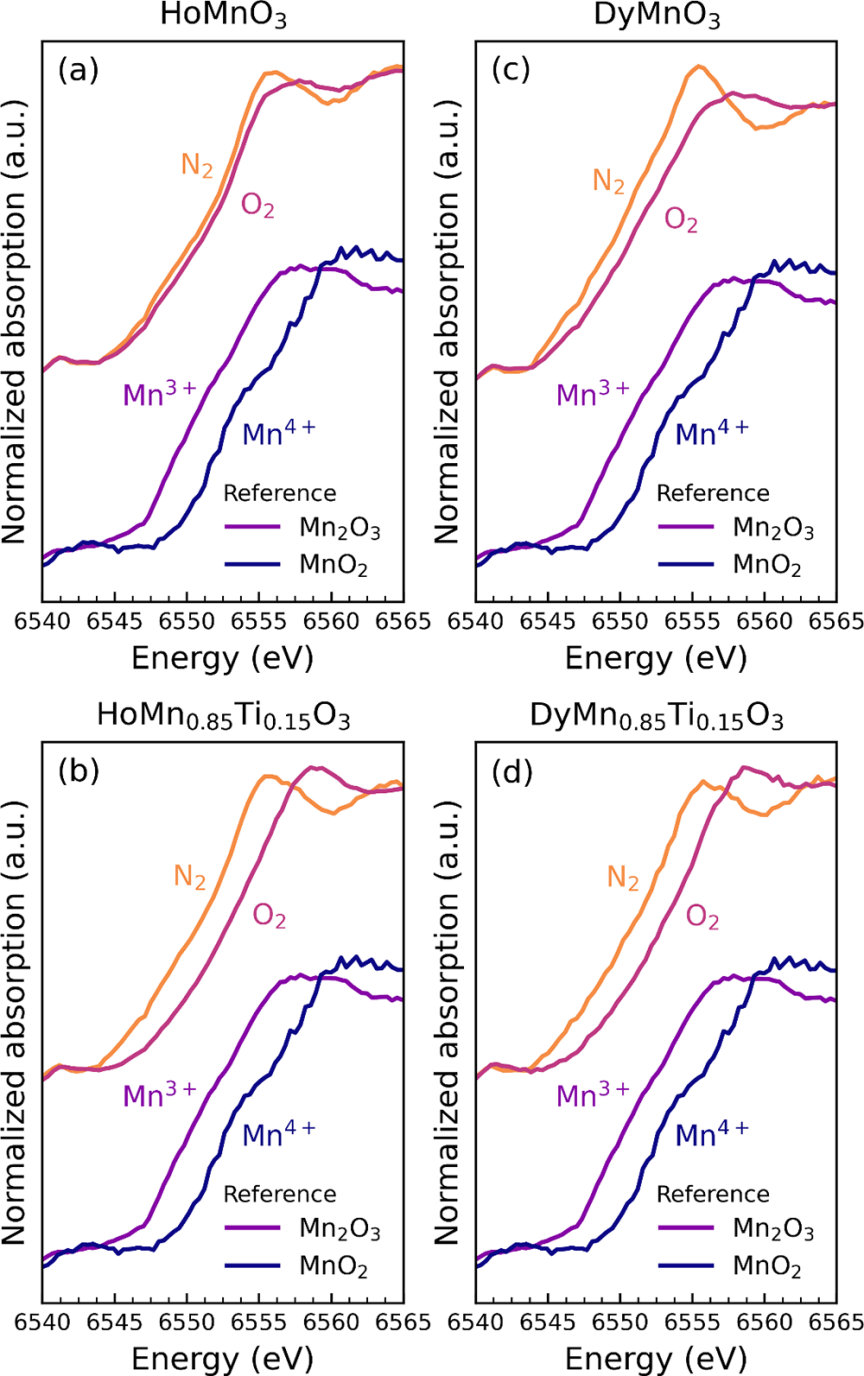
Normalized XANES spectra at the Mn K-edge of (a) HoMnO_3_, (b) HoMn_0.85_Ti_0.15_O_3_, (c) DyMnO_3_, and (d) DyMn_0.85_Ti_0.15_O_3_ before (N_2_) and after oxidation (O_2_) at 300 °C. The spectra of Mn_2_O_3_ and MnO_2_ are shown as references.

All samples display a partial oxidation of Mn, from Mn^3+^ to Mn^4+^, when exposed to O_2_ atmosphere. Normalized X-ray absorption near-edge structure (XANES) spectra of the Mn K-edge measured before and after oxidation at 300 °C are shown in Figure 4 and at other temperatures in Supplementary Figures S2–S4. The spectra collected after oxidation, noted “O_2_” in Figure 4, are all shifted toward higher energies than the spectra measured in N_2_. As shown by the Mn_2_O_3_ and MnO_2_ references, with the Mn K-edge of MnO_2_ being at higher energies than that of Mn_2_O_3_, this shift indicates an oxidation process from Mn^3+^ to Mn^4+^, which is the expected charge compensation mechanism upon absorption of oxygen. The Ti-doped samples in panels (b) and (d) display a shift with greater magnitude than the undoped samples, indicating that a larger amount of Mn^3+^ has been oxidized to Mn^4+^. This corresponds well with the larger oxygen content that Ti-doped samples possess at 300 °C (Figure 3). The spectra of HoMnO_3_ in (a) show the smallest change in absorption edge energies after being exposed to O_2_, as this composition shows low OSC (δ ≈ 0.06) at this temperature.

**Figure 5:**
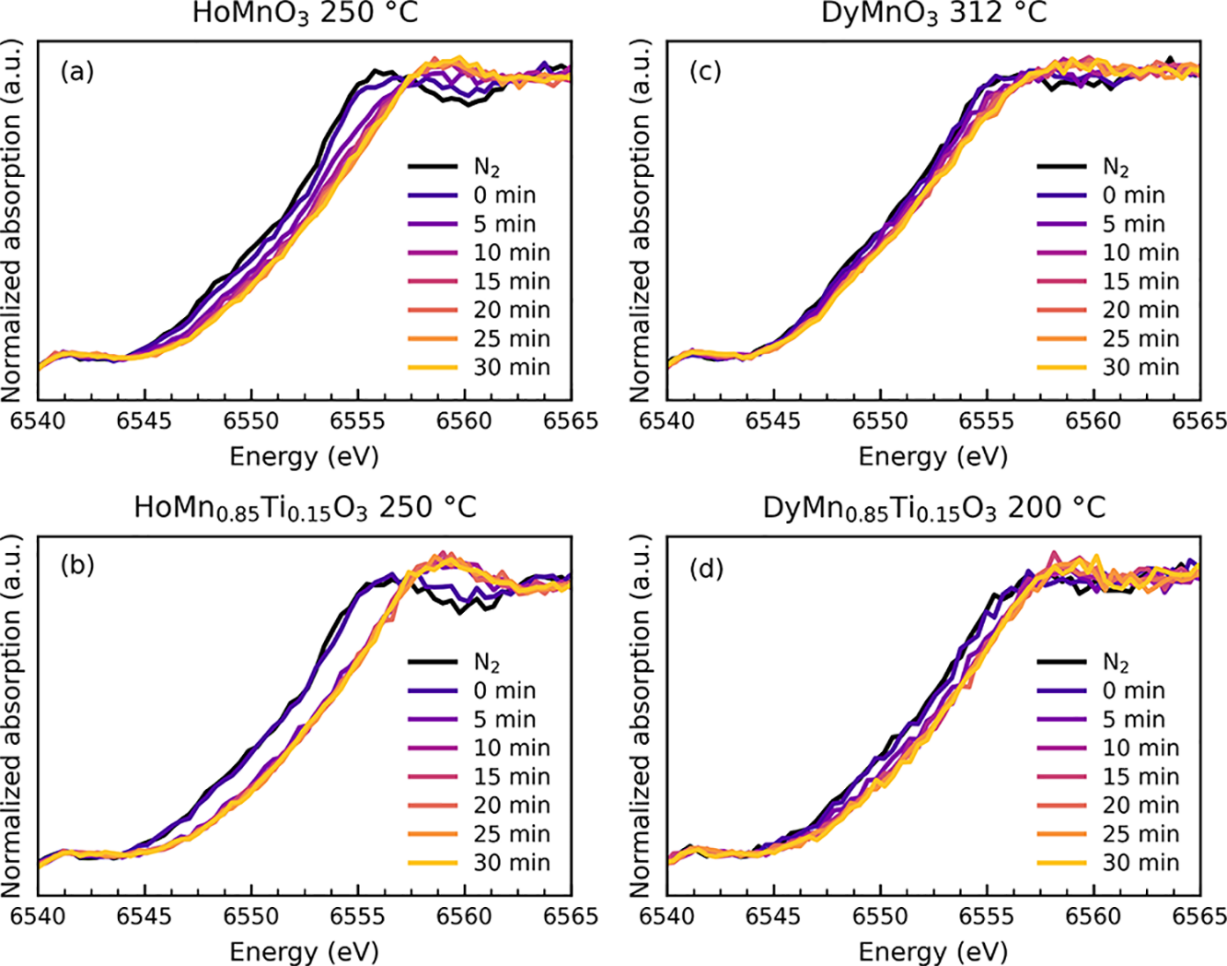
Normalized time-resolved XANES collected upon in situ switching of atmosphere from N_2_ to O_2_ for (a) HoMnO_3_, (b) HoMn_0.85_Ti_0.15_O_3_, (c) DyMnO_3_, and (d) DyMn_0.85_Ti_0.15_O_3_ at different temperatures. Measurements done in O_2_ are labeled as the time after switching from N_2_ to O_2_ atmosphere.

XANES spectra measured during oxidation after in situ switching of the atmosphere from N_2_ to O_2_ are shown in Figure 5. The Mn K-edge position shifts progressively to higher energies with time, with the Ti-doped samples reaching the highest oxidation state faster than the undoped samples. The largest shifts in edge position occur during the first 10 min for all samples. Temperatures for each sample were chosen where the oxidation progresses at a speed allowing good quality spectra to be collected within a reasonable time.

**Figure 6:**
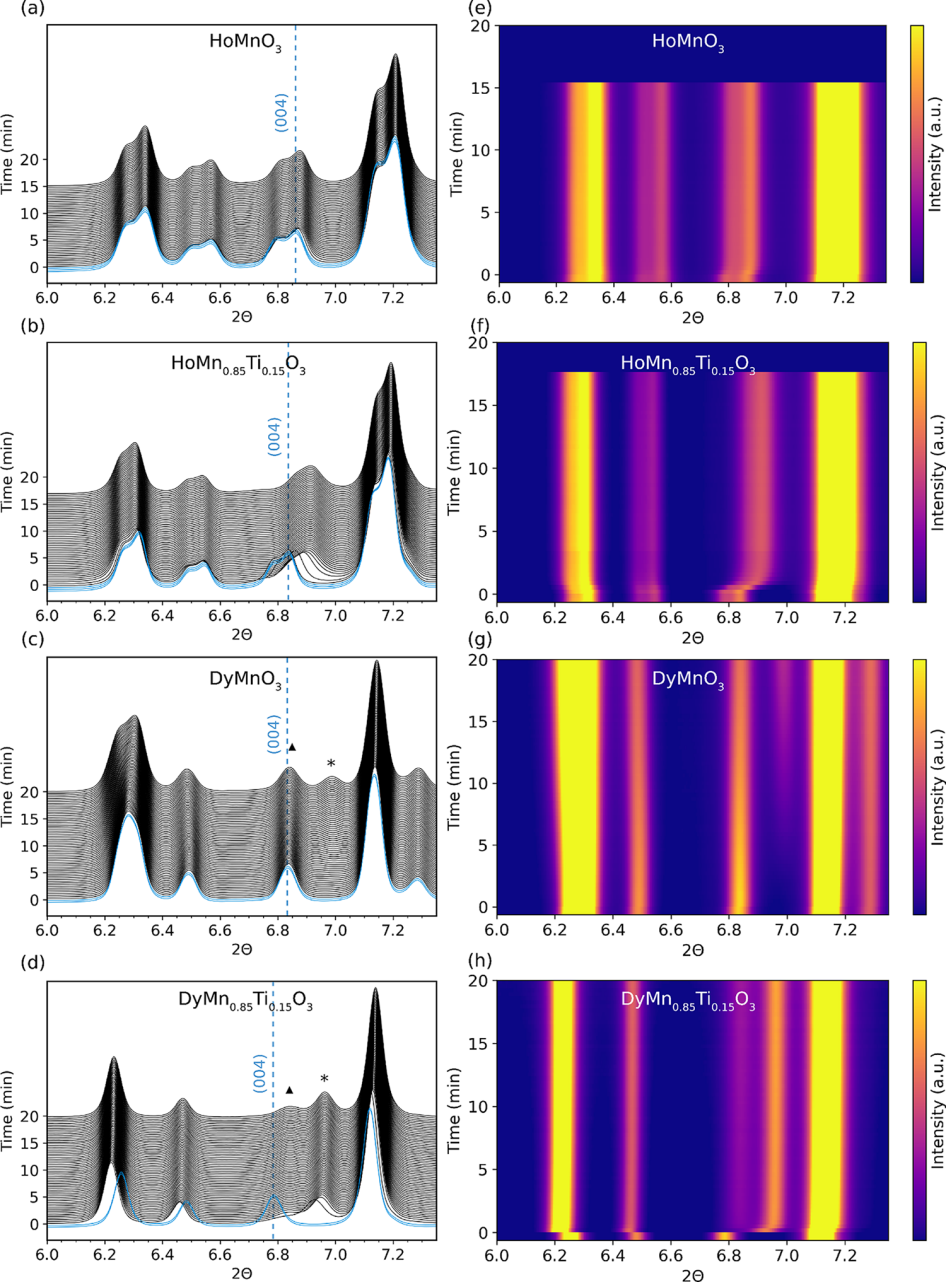
X-ray diffractograms and 2D contour plots of (a, e) HoMnO_3_, (b, f) HoMn_0.85_Ti_0.15_O_3_, (c, g) DyMnO_3_, and (d, h) DyMn_0.85_Ti_0.15_O_3_ measured at 300 °C as a function of time after in situ switching of atmosphere from N_2_ (blue) to O_2_ (black). The baseline intensity at 6.0° 2θ in (a–d) indicates the time in min after switching from N_2_ to O_2_ purge gas. Vertical dashed lines (blue) indicate the initial position of the (0 0 4) reflection measured in N_2_. The triangles (▲) indicate the (1 0 10) reflection, and the asterisks (*) indicate the (0 0 12) reflection, both characteristic for the *R*3*c* space group.

Synchrotron X-ray diffractograms measured at 300 °C upon in situ switching of atmosphere from N_2_ to O_2_ are shown in Figure 6, depicted as waterfall plots in (a)–(d) and as 2D contour plots in (e)–(h). Measurements at other temperatures can be found in Figures S6–S11. All samples show shifts in the position of the (0 0 4) reflection at ∼6.8° 2θ toward higher angles during oxidation, reflecting a contraction of the *c* axis when interstitial oxygen enters MnO planes. This contraction is attributed to partial rectification of the tilting of the MnO_5_ bipyramids and the smaller radius of Mn^4+^ which is charge compensating interstitial O^2–^. In the Dy-containing samples, shown in panels (c, d) and (g, h), the single (0 0 4) reflection of the *P*6_3_*cm* structure (marked with triangles ▲) is replaced by the two (1 0 10) and (0 0 12) reflections (marked with asterisks *) of the new structure with space group *R*3*c* during oxidation, respectively. The appearance of these two reflections demonstrates the transition to the oxygen-rich structure with space group *R*3*c* (δ > 0.28), where the *c* axis is tripled compared to the stoichiometric structure with space group *P*6_3_*cm*.^24,26,40^ Another observable in Figure 6 is the faster oxidation process for the Ti-doped samples compared to the undoped samples; both HoMn_0.85_Ti_0.15_O_3_ and DyMn_0.85_Ti_0.15_O_3_ stabilize within 2 min, while DyMnO_3_ needs over 15 min to fully oxidize. The HoMnO_3_ sample does not oxidize significantly at 300 *°*C, as shown in the TGA measurements in Figure 3.

**Figure 7:**
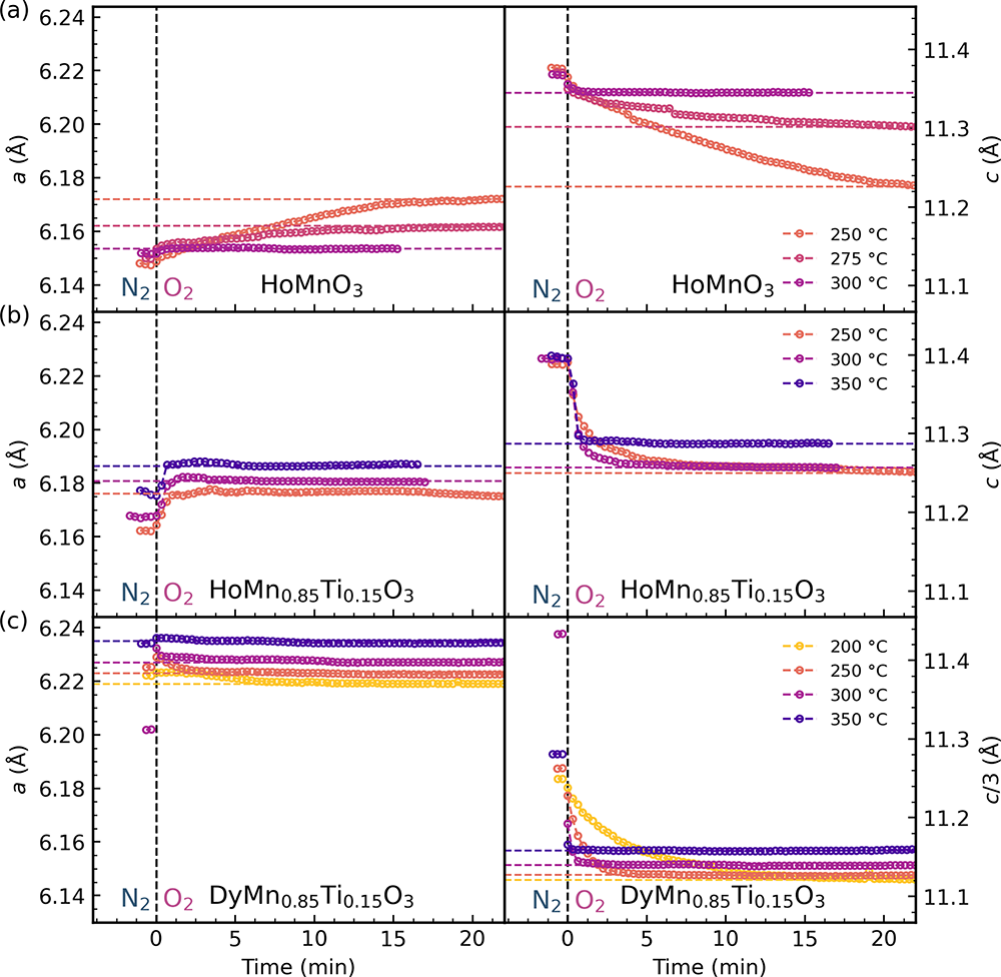
Refined lattice parameters *a* and *c* as a function of time after switching from N_2_ to O_2_ atmosphere for (a) HoMnO_3_, (b) HoMn_0.85_Ti_0.15_O_3_, and (c) DyMn_0.85_Ti_0.15_O_3_. For DyMn_0.85_Ti_0.15_O_3_, which were refined within the *R*3*c* space group, *c*/*a* is shown for easier comparison with the refinements within the *P*6_3_*cm* space group.

Changes in lattice parameters are proposed to be closely linked to the oxygen stoichiometry through chemical expansion,^49^ through which the kinetics of oxidation can be investigated indirectly.^50^ Lattice parameters of HoMnO_3_, HoMn_0.85_Ti_0.15_O_3_, and DyMn_0.85_Ti_0.15_O_3_ as a function of time after switching from N_2_ to O_2_ atmosphere at different temperatures are shown in Figure 7, with measurements of DyMn_0.85_Ti_0.15_O_3_ at additional temperatures included in Figure S12. For all samples, the oxidation process is faster with increasing temperatures, as expected. The net expansion and contraction of the lattice parameters decrease with increasing temperature, as the materials oxidize less at higher temperatures. This is most significant for HoMnO_3_ (Figure 7a), with almost no change in lattice parameters above 300 °C where most of the excess oxygen has already desorbed, as measured by TGA (Figure 3). Both undoped (Figure 7a) and Ti-doped HoMnO_3_ (Figure 7b) show an expansion in the *a* parameter during oxidation, while Ti-doped DyMnO_3_ (Figure 7c) shows initially an expansion and then a contraction as the sample oxidizes further. This is in accordance with previous work on Ti-doped DyMnO_3_.^40^ The equilibration time for the oxidation decreases with increasing R cation size and with Ti-doping; at 250 °C, HoMnO_3_ takes over 20 min to reach equilibrium, while Ti-doped HoMnO_3_ and DyMnO_3_ each take ∼15 and ∼5 min, respectively. At 350 °C, both Ti-doped samples oxidize very rapidly, with Ti-doped HoMnO_3_ reaching equilibrium in <1 min, and Ti-doped DyMnO_3_ in a few seconds. Lattice parameters for undoped DyMnO_3_ are not shown due to unstable Pawley refinements of the data collected under the gradual transition between *P*6_3_*cm* and *R*3*c* during oxidation.

## Discussion

### Ionic Radii of R

The oxidation kinetics of hexagonal manganites improve from Ho^3+^ (1.015 Å, C.N = 7^51^) to the larger Dy^3+^ (1.027 Å), as seen from the X-ray diffractograms (Figure 6) and lattice parameters (Figure 7). A direct comparison of the kinetics between undoped HoMnO_3_ and DyMnO_3_ is not straightforward as these materials oxidize at different temperatures. The relationship between unit cell volume and oxygen diffusion rate has previously been reported for R_0.25_Y_0.75_MnO_3_, which showed improved oxygen absorption and release rates with larger R^3+^ cations than Dy^3+^.^38^ We hypothesize that the expansion of the unit cell caused by the larger R^3+^ cations, which necessarily increases the interatomic distances in the Mn–O sublattice, reduces the electrostatic repulsion between planar oxygen ions and the migrating O_i_ ion in the interstitialcy mechanism.^28^ It is not known if the same structural changes also favor the surface exchange reaction where O_2_ molecules adsorb and split, but empirically we observe that unit cell expansion, particularly in the *ab*-plane, improves the kinetics of oxygen absorption and desorption.

### Ti Donor Doping

As evident from both TGA (Figure 3) and time-resolved in situ measurements (Figures 5–7), there is a significant increase in the oxidation rate upon doping with 15% Ti, reducing the time for full oxidation by an order of magnitude. In the hexagonal *P*6_3_*cm* structure, each MnO_5_ bipyramid in the *ab*-layer is slightly tilted in a pattern of trimers, and the R^3+^ cations are displaced along the *c* axis.^52^ Ti^4+^ ions on the Mn^3+^ sites have been shown to stabilize the high symmetry *P*6_3_/*mmc* phase with untilted bipyramids and nondisplaced R to lower temperatures.^53^ We hypothesize that the less distorted structure, comparable to the high-symmetry phase, also favors faster oxidation, as higher symmetry in general promotes ionic conductivity and because of the ion migration mechanism in these materials, as discussed below. We hypothesize that the presence of Ti may also enhance the ecatalytic splitting of O_2_ molecules in the surface exchange reaction, although this cannot be proven based on the literature or the present data. HoMn_0.85_Ti_0.15_O_3_ and DyMn_0.85_Ti_0.15_O_3_ display larger *a* lattice parameters compared to their undoped counterparts both before (Table 1) and after oxidation (Figure 7), resulting in an expanded *ab*-plane similar to the effect of larger R^3+^.

**Figure 8:**
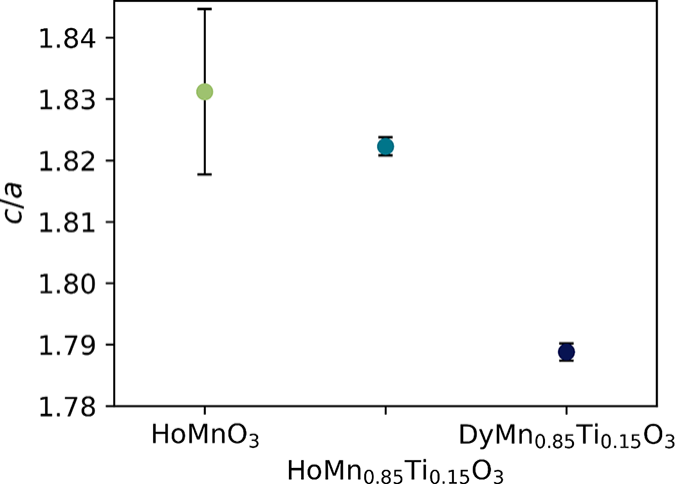
Average *c*/*a* of oxidized HoMnO_3_, HoMn_0.85_Ti_0.15_O_3_, and DyMn_0.85_Ti_0.15_O_3_. For DyMn_0.85_Ti_0.15_O_3_, *c*/3 was used instead of *c* for easier comparison between the *R*3*c* and *P*6_3_*cm* structures.

The average *c*/*a* ratios determined from the refined lattice parameters of oxidized samples (Figure 7) are shown in Figure 8, showing an evolution toward, and beyond, the value of 1.81 found at the *T*_C_ of YMnO_3_.^52,54^ The change of structure toward the *P*6_3_/*mmc* structure as induced by Ti-doping is also significant because it implies a reduction of the distortion mode amplitude of the *K*_3_ distortion mode driving the transition to *P*6_3_*cm*.^52,54−56^ In real space, the ionic displacement vectors resulting from freezing in the *K*_3_ mode have strong similarities to those describing the transition state of the interstitialcy migration mechanism for O_i_.^28^ While the connection between phonons and ionic migration is generally poorly understood,^57,58^ we hypothesize that Ti doping-induced partial melting of the *K*_3_ mode, pushing the material toward higher symmetry, also enhances the kinetics of oxygen bulk transport.

The absence of thermal hysteresis during heating and cooling, the increased maximum OSC, and the close-to-zero decrease in OSC upon increasing cooling rate found through TGA (Figure 3) show that Ti-doped RMnO_3_ can be utilized for oxygen absorption at faster rates than undoped materials. As demonstrated here (Figure 3) and in previous studies,^40,42^ interstitial oxygen is stabilized toward higher temperatures for Ti-doped samples. Compared to undoped samples, Ti-doping enables pressure swing absorption at higher temperatures where the kinetics of oxidation and reduction will be much faster.

### Surface Exchange Rate

The significant contraction along the *c* axis induced by the incorporation of interstitial oxygen can be used as a measure of the oxygen content, and thus the oxygen surface exchange rate can be inferred from the transient lattice parameters during oxidation after switching from N_2_ to O_2_ atmosphere. We investigated the kinetics of oxidation by fitting refined lattice parameters to the model developed by Moreno et al.^50^ For sufficiently small sample dimensions, usually below a few tens of micrometers, the surface exchange reaction is the rate-limiting step for oxygen incorporation.^59,60^ Using the contraction of the *c* parameter as a measure of the degree of oxidation of the material, a direct correspondence between unit cell parameter and concentration of oxygen is assumed. The defect concentration at a given time, *c*(*t*), will then follow an exponential equation:
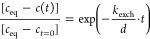
1where *c*_eq_ is the equilibrium concentration, *c*_*t*=0_ is the start concentration, *k*_exch_ is the surface exchange rate coefficient, and *d* is the average size of the material sample, e.g., film thickness, or in our case half the particle size. The surface exchange rate *k* can now be estimated by fitting the refined *c* lattice parameters presented in Figures 7 and S12 to eq 1.

**Figure 9:**
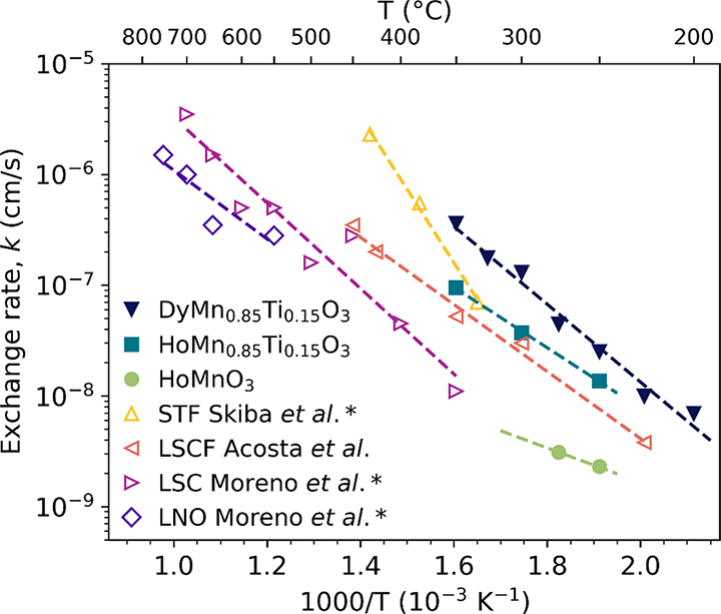
Temperature dependence of oxygen surface exchange rates *k* upon changing atmosphere from N_2_ to O_2_. Surface exchange rates for SrTi_0.65_Fe_0.35_O_2.825+δ_ from Skiba et al.,^63^ (La_0.60_Sr_0.40_)_0.95_(Co_0.20_Fe_0.80_)O_3−δ_ from Acosta et al.,^66^ and La_0.6_Sr_0.4_CoO_3-δ_ and LaNiO_3−δ_ from Moreno et al.^16^ are included for comparison purposes. Exchange rates marked with an asterisk (*) were measured using *p*_O2_ = 0.21.

The calculated oxygen surface exchange rates for HoMnO_3_, HoMn_0.85_Ti_0.15_O_3_, and DyMn_0.85_Ti_0.15_O_3_ are presented in Figure 9. The surface exchange rates increase with increasing R cation size, and with Ti-doping, and the rates of the Ti-doped samples are comparable to what has been reported for perovskites and other oxides at much higher temperatures.^16,50,59,61−66^ The surface exchange rate for HoMnO_3_ at 300 °C is not included, as very little oxidation happens in this sample at higher temperatures, and the resulting calculated exchange rate is therefore unreliable.

The activation energies calculated from the surface exchange rates for each sample were found to be 0.54 eV for HoMn_0.85_Ti_0.15_O_3_ and 0.68 eV for DyMn_0.85_Ti_0.15_O_3_. The activation energy for HoMnO_3_ was not calculated as this sample had only two data points, which would result in a high uncertainty in the calculated value. These activation energies are slightly lower than the typical values reported for hexagonal Pr_0.05_Y_0.95_MnO_3_ (0.8–0.9 eV)^67^ and for perovskites (∼0.7–1.1 eV).^16,63,68,69^ From Figure 7, it can be observed that DyMn_0.85_Ti_0.15_O_3_ displays an initial expansion followed by a contraction in the *ab*-plane during oxidation, which differs from the other samples that only show expansion. One possible explanation for this is that the electrostatic attraction between the Ti^4+^ present in the already expanded *ab*-plane and the negatively charged O_i_ entering the lattice is larger than the electrostatic repulsion between planar oxygen and O_i_, causing a contraction along the *a* axis and an increase in stability of interstitial oxygen in the structure compared to the undoped samples. Substituting YMnO_3_ with larger R cations such as Sm^3+^ can stabilize the oxidized phase and thus lower the oxygen release rate.^38^ The TGA data in Figure 3 show no sign that the stronger bond between Ti^4+^ and O_i_ is lowering the rate of oxygen release, and the lack of thermal hysteresis confirms that the kinetics of reduction is still fast.

For hexagonal manganites to be used for CLAS, both the OSC and the kinetics of oxidation and reduction must be sufficiently high. While the OSC of hexagonal manganites has become competitive compared to other material classes, future work on RMnO_3_ for CLAS should focus on improving the oxidation kinetics. It is now established that larger R^3+^ improves both the oxidation rate and OSC, and large rare earth dopants should be included in future studies. The addition of Ti^4+^ affects both the OSC and the kinetics of oxidation positively, but it also limits these materials to pressure swing applications, as the broad temperature interval for oxidation and reduction is impractical for temperature swing processes. Other aliovalent dopants may also be beneficial; e.g., Zr^4+^ on the Y-sublattice has displayed similar effects as Ti^4+^ on the Mn-sublattice for the OSC and thermal stability of O_i_,^43^ but its effect on oxidation kinetics is unknown.

## Conclusions

The kinetics of oxidation of nanocrystalline *R*MnO_3_ has been studied by thermogravimetric analysis and HT-XRD upon in situ switching of atmosphere from N_2_ to O_2_. The rate of oxidation increases for larger R^3+^ cations and with 15% Ti^4+^-doping on the Mn sublattice. The OSC of Ti-doped samples is insensitive to the rate of cooling in O_2_ atmosphere, signifying very fast kinetics of oxidation. Changes in lattice parameters inferred from HT-XRD showed that the oxidation times decrease substantially when Ho^3+^ is replaced by the larger Dy^3+^ and even more when substituting 15% Ti^4+^ on the Mn sublattice. The oxidation of DyMn_0.85_Ti_0.15_O_3_, which showed the fastest kinetics, was completed within only a few seconds at 350 °C in O_2_ atmosphere. Faster oxidation kinetics were correlated with expansion of the *ab*-plane caused by the larger ionic radius of Dy^3+^ and the Ti^4+^-doping-induced partial rectification of the tilted MnO_5_ trigonal bipyramids, which also results in a smaller *c*/*a* ratio. The change in structure toward the high symmetry *P*6_3_/*mmc* phase is also hypothesized to improve the kinetics of oxygen transport. The changes in lattice parameters were used to calculate surface exchange rates, which also showed improvement with larger rare earth cations and with Ti doping.

## Supporting Information Available

Additional data from XRD and XANES collected under ambient conditions and in situ upon gas shift and refined lattice parameters as a function of time upon gas shift (PDF)

## References

[ref1] Kirschner M. J.; Alekseev A.; Dowy S.; Grahl M.; Jansson L.; Keil P.; Lauermann G.; Meilinger M.; Schmehl W.; Weckler H.; Windmeier C. Oxygen. In Ullmann’s Encyclopedia of Industrial Chemistry; Wiley-VCH Verlag GmbH & Co. KGaA: Weinheim, Germany, 2017; pp 1–32.

[ref2] Wu F.; Argyle M. D.; Dellenback P. A.; Fan M. Progress in O2 Separation for Oxy-Fuel Combustion–A Promising Way for Cost-Effective CO2 Capture: A Review. Prog. Energy Combust. Sci. 2018, 67, 188–205. 10.1016/j.pecs.2018.01.004.

[ref3] Häring H. W.; Ahner C.; Belloni A. Industrial Gases Processing; Wiley, 2007.

[ref4] Smith A.; Klosek J. A Review of Air Separation Technologies and Their Integration with Energy Conversion Processes. Fuel Process. Technol. 2001, 70, 115–134. 10.1016/S0378-3820(01)00131-X.

[ref5] Zhou C.; Shah K.; Moghtaderi B. Techno-Economic Assessment of Integrated Chemical Looping Air Separation for Oxy-Fuel Combustion: An Australian Case Study. Energy Fuels 2015, 29, 2074–2088. 10.1021/ef5022076.

[ref6] Moghtaderi B. Application of Chemical Looping Concept for Air Separation at High Temperatures. Energy Fuels 2010, 24, 190–198. 10.1021/ef900553j.

[ref7] Tang M.; Xu L.; Fan M. Progress in Oxygen Carrier Development of Methane-Based Chemical-Looping Reforming: A Review. Appl. Energy 2015, 151, 143–156. 10.1016/j.apenergy.2015.04.017.

[ref8] Zhu X.; Li K.; Neal L.; Li F. Perovskites as Geo-Inspired Oxygen Storage Materials for Chemical Looping and Three-Way Catalysis: A Perspective. ACS Catal. 2018, 8, 8213–8236. 10.1021/acscatal.8b01973.

[ref9] Demont A.; Abanades S.; Beche E. Investigation of Perovskite Structures as Oxygen-Exchange Redox Materials for Hydrogen Production from Thermochemical Two-Step Water-Splitting Cycles. J. Phys. Chem. C 2014, 118, 12682–12692. 10.1021/jp5034849.

[ref10] Krzystowczyk E.; Wang X.; Dou J.; Haribal V.; Li F. Substituted SrFeO_3_ as Robust Oxygen Sorbents for Thermochemical Air Separation: Correlating Redox Performance with Compositional and Structural Properties. Phys. Chem. Chem. Phys. 2020, 22, 8924–8932. 10.1039/d0cp00275e. 32292966

[ref11] Vieten J.; Bulfin B.; Senholdt M.; Roeb M.; Sattler C.; Schmücker M. Redox Thermodynamics and Phase Composition in the System SrFeO_3−δ_ — SrMnO_3−δ_. Solid State Ionics 2017, 308, 149–155. 10.1016/j.ssi.2017.06.014.

[ref12] Bulfin B.; Vieten J.; Starr D. E.; Azarpira A.; Zachäus C.; Hävecker M.; Skorupska K.; Schmücker M.; Roeb M.; Sattler C. Redox Chemistry of CaMnO 3 and Ca 0.8 Sr 0.2 MnO 3 Oxygen Storage Perovskites. J. Mater. Chem. A 2017, 5, 7912–7919. 10.1039/C7TA00822H.

[ref13] Krzystowczyk E.; Haribal V.; Dou J.; Li F. Chemical Looping Air Separation Using a Perovskite-Based Oxygen Sorbent: System Design and Process Analysis. ACS Sustainable Chem. Eng. 2021, 9, 12185–12195. 10.1021/acssuschemeng.1c03612.

[ref14] Xu X.; Wang W.; Zhou W.; Shao Z. Recent Advances in Novel Nanostructuring Methods of Perovskite Electrocatalysts for Energy-Related Applications. Small Methods 2018, 2, 1800071 10.1002/smtd.201800071.

[ref15] Xu X.; Su C.; Shao Z. Fundamental Understanding and Application of Ba_0.5_Sr_0.5_Co_0.8_Fe_0.2_O_3−δ_Perovskite in Energy Storage and Conversion: Past, Present, and Future. Energy Fuels 2021, 35, 13585–13609. 10.1021/acs.energyfuels.1c02111.

[ref16] Moreno R.; Zapata J.; Roqueta J.; Bagués N.; Santiso J. Chemical Strain and Oxidation-Reduction Kinetics of Epitaxial Thin Films of Mixed Ionic-Electronic Conducting Oxides Determined by X-Ray Diffraction. J. Electrochem. Soc. 2014, 161, F3046–F3051. 10.1149/2.0091411jes.

[ref17] Cai G.; Luo C.; Zheng Y.; Cao D.; Luo T.; Li X.; Wu F.; Zhang L. BaCoO_3−δ_ Perovskite-Type Oxygen Carrier for Chemical Looping Air Separation, Part I: Determination of Oxygen Non-Stoichiometry and Cyclic Stability of Oxygen Carrier. Sep. Purif. Technol. 2022, 302, 121972 10.1016/j.seppur.2022.121972.

[ref18] Lein H.; Wiik K.; Grande T. Kinetic Demixing and Decomposition of Oxygen Permeable Membranes. Solid State Ionics 2006, 177, 1587–1590. 10.1016/j.ssi.2006.03.001.

[ref19] Dou J.; Krzystowczyk E.; Wang X.; Robbins T.; Ma L.; Liu X.; Li F. A- and B-site Codoped SrFeO_3_ Oxygen Sorbents for Enhanced Chemical Looping Air Separation. ChemSusChem 2020, 13, 385–393. 10.1002/cssc.201902698. 31710175

[ref20] Bulfin B.; Lapp J.; Richter S.; Gubàn D.; Vieten J.; Brendelberger S.; Roeb M.; Sattler C. Air Separation and Selective Oxygen Pumping via Temperature and Pressure Swing Oxygen Adsorption Using a Redox Cycle of SrFeO_3_ Perovskite. Chem. Eng. Sci. 2019, 203, 68–75. 10.1016/j.ces.2019.03.057.

[ref21] Vieten J.; Bulfin B.; Call F.; Lange M.; Schmücker M.; Francke A.; Roeb M.; Sattler C. Perovskite Oxides for Application in Thermochemical Air Separation and Oxygen Storage. J. Mater. Chem. A 2016, 4, 13652–13659. 10.1039/C6TA04867F.

[ref22] Remsen S.; Dabrowski B. Synthesis and Oxygen Storage Capacities of Hexagonal Dy 1– x Y x MnO 3+δ. Chem. Mater. 2011, 23, 3818–3827. 10.1021/cm2006956.

[ref23] Remsen S.; Dabrowski B.; Chmaissem O.; Mais J.; Szewczyk A. Synthesis and Oxygen Content Dependent Properties of Hexagonal DyMnO_3+δ_. J. Solid State Chem. 2011, 184, 2306–2314. 10.1016/j.jssc.2011.06.037.

[ref24] Abughayada C.; Dabrowski B.; Kolesnik S.; Brown D. E.; Chmaissem O. Characterization of Oxygen Storage and Structural Properties of Oxygen-Loaded Hexagonal RMnO_3+δ_ (R = Ho, Er, and Y). Chem. Mater. 2015, 27, 6259–6267. 10.1021/acs.chemmater.5b01817.

[ref25] Świerczek K.; Klimkowicz A.; Nishihara K.; Kobayashi S.; Takasaki A.; Alanizy M.; Kolesnik S.; Dabrowski B.; Seong S.; Kang J. Oxygen Storage Properties of Hexagonal HoMnO_3+δ_. Phys. Chem. Chem. Phys. 2017, 19, 19243–19251. 10.1039/c7cp03556j. 28702623

[ref26] Abughayada C.; Dabrowski B.; Avdeev M.; Kolesnik S.; Remsen S.; Chmaissem O. Structural, Magnetic, and Oxygen Storage Properties of Hexagonal Dy_1–x_Y_x_MnO_3+δ_. J. Solid State Chem. 2014, 217, 127–135. 10.1016/j.jssc.2014.05.017.

[ref27] Selbach S. M.; Løvik A. N.; Bergum K.; Tolchard J. R.; Einarsrud M. A.; Grande T. Crystal Structure, Chemical Expansion and Phase Stability of HoMnO_3_ at High Temperature. J. Solid State Chem. 2012, 196, 528–535. 10.1016/j.jssc.2012.07.024.

[ref28] Skjærvø S. H.; Wefring E. T.; Nesdal S. K.; Gaukås N. H.; Olsen G. H.; Glaum J.; Tybell T.; Selbach S. M. Interstitial Oxygen as a Source of P-Type Conductivity in Hexagonal Manganites. Nat. Commun. 2016, 7, 13745 10.1038/ncomms13745. 27924812 PMC5150987

[ref29] van Roosmalen J. A. M.; Cordfunke E. H. P. The Defect Chemistry of LaMnO_3±δ_ – 4. Defect Model for LaMnO_3+δ_. J. Solid State Chem. 1994, 110, 109–112. 10.1006/jssc.1994.1143.

[ref30] Griffin S. M.; Reidulff M.; Selbach S. M.; Spaldin N. A. Defect Chemistry as a Crystal Structure Design Parameter: Intrinsic Point Defects and Ga Substitution in InMnO_3_. Chem. Mater. 2017, 29, 2425–2434. 10.1021/acs.chemmater.6b04207.

[ref31] Kotomin E. A.; Mastrikov Y. A.; Heifets E.; Maier J. Adsorption of Atomic and Molecular Oxygen on the LaMnO_3_ (001) Surface: Ab Initio Supercell Calculations and Thermodynamics. Phys. Chem. Chem. Phys. 2008, 10, 4644–4649. 10.1039/b804378g. 18665314

[ref32] Polfus J. M.; Yildiz B.; Tuller H. L. Origin of Fast Oxide Ion Diffusion along Grain Boundaries in Sr-Doped LaMnO_3_. Phys. Chem. Chem. Phys. 2018, 20, 19142–19150. 10.1039/c8cp02443j. 29975388

[ref33] Carrasco J.; Illas F.; Lopez N.; Kotomin E. A.; Zhukovskii Y. F.; Evarestov R. A.; Mastrikov Y. A.; Piskunov S.; Maier J. First-Principles Calculations of the Atomic and Electronic Structure of F Centers in the Bulk and on the (001) Surface of SrTiO_3_. Phys. Rev. B 2006, 73, 064106 10.1103/PhysRevB.73.064106.

[ref34] Grande T.; Tolchard J. R.; Selbach S. M. Anisotropic Thermal and Chemical Expansion in Sr-Substituted LaMnO 3+δ: Implications for Chemical Strain Relaxation. Chem. Mater. 2012, 24, 338–345. 10.1021/cm2030608.

[ref35] Klimkowicz A.; Cichy K.; Chmaissem O.; Dabrowski B.; Poudel B.; Świerczek K.; Taddei K. M.; Takasaki A. Reversible Oxygen Intercalation in Hexagonal Y_0.7_Tb_0.3_MnO_3+δ_ : Toward Oxygen Production by Temperature-Swing Absorption in Air. J. Mater. Chem. A 2019, 7, 2608–2618. 10.1039/c8ta09235d.

[ref36] Klimkowicz A.; Świerczek K.; Kobayashi S.; Takasaki A.; Allahyani W.; Dabrowski B. Improvement of Oxygen Storage Properties of Hexagonal YMnO3+δ by Microstructural Modifications. J. Solid State Chem. 2018, 258, 471–476. 10.1016/j.jssc.2017.10.037.

[ref37] Cichy K.; Świerczek K.; Jarosz K.; Klimkowicz A.; Marzec M.; Gajewska M.; Dabrowski B. Towards Efficient Oxygen Separation from Air: Influence of the Mean Rare-Earth Radius on Thermodynamics and Kinetics of Reactivity with Oxygen in Hexagonal Y_1-x_R_x_MnO_3+δ_. Acta Mater. 2021, 205, 116544 10.1016/j.actamat.2020.116544.

[ref38] Otomo M.; Hasegawa T.; Asakura Y.; Yin S. Remarkable Effects of Lanthanide Substitution for the Y-Site on the Oxygen Storage/Release Performance of YMnO_3+δ_. ACS Appl. Mater. Interfaces 2021, 13, 31691–31698. 10.1021/acsami.1c06880. 34185497

[ref39] Cichy K.; Zając M.; Świerczek K. Evaluation of Applicability of Nd- and Sm-Substituted Y_1-x_R_x_MnO_3+δ_ in Temperature Swing Absorption for Energy-Related Technologies. Energy 2022, 239, 122429 10.1016/j.energy.2021.122429.

[ref40] Danmo F. H.; Williamson B. A. D.; Småbråten D. R.; Gaukås N. H.; Østli E. R.; Grande T.; Glaum J.; Selbach S. M. Oxygen Absorption in Nanocrystalline H-RMnO_3_ (R = Y, Ho, Dy) and the Effect of Ti Donor Doping. Chem. Mater. 2023, 35, 5764 10.1021/acs.chemmater.3c00189.

[ref41] Klimkowicz A.; Hashizume T.; Cichy K.; Tamura S.; Świerczek K.; Takasaki A.; Motohashi T.; Dabrowski B. Oxygen Separation from Air by the Combined Temperature Swing and Pressure Swing Processes Using Oxygen Storage Materials Y_1–x_(Tb/Ce)_x_MnO_3+δ_. J. Mater. Sci. 2020, 55, 15653–15666. 10.1007/s10853-020-05158-5.

[ref42] Levin I.; Krayzman V.; Vanderah T. A.; Tomczyk M.; Wu H.; Tucker M. G.; Playford H. Y.; Woicik J. C.; Dennis C. L.; Vilarinho P. M. Oxygen-Storage Behavior and Local Structure in Ti-Substituted YMnO_3_. J. Solid State Chem. 2017, 246, 29–41. 10.1016/j.jssc.2016.10.029.

[ref43] Moreno Botello Z. L.; Montenegro A.; Grimaldos Osorio N.; Huvé M.; Pirovano C.; Småbråten D. R.; Selbach S. M.; Caneiro A.; Roussel P.; Gauthier G. H. Pure and Zr-Doped YMnO_3+δ_ as a YSZ-Compatible SOFC Cathode: A Combined Computational and Experimental Approach. J. Mater. Chem. A 2019, 7, 18589–18602. 10.1039/c9ta04912f.

[ref44] Holstad T. S.; Evans D. M.; Ruff A.; Småbråten D. R.; Schaab J.; Tzschaschel C.; Yan Z.; Bourret E.; Selbach S. M.; Krohns S.; Meier D. Electronic Bulk and Domain Wall Properties in B -Site Doped Hexagonal ErMnO_3_. Phys. Rev. B 2018, 97, 085143 10.1103/PhysRevB.97.085143.

[ref45] Momma K.; Izumi F. VESTA 3 for Three-Dimensional Visualization of Crystal, Volumetric and Morphology Data. J. Appl. Crystallogr. 2011, 44, 1272–1276. 10.1107/S0021889811038970.

[ref46] Bergum K.; Okamoto H.; Fjellvåg H.; Grande T.; Einarsrud M. A.; Selbach S. M. Synthesis, Structure and Magnetic Properties of Nanocrystalline YMnO_3_. Dalton Trans. 2011, 40, 7583–7589. 10.1039/c1dt10536a. 21701735

[ref47] Coelho A. A. Topas Academic: General Profile and Structure Analysis Software for Powder Diffraction Data; Bruker AXS: Karlsruhe, Germany, 2004.

[ref48] Newville M. L. An Analysis Package for XAFS and Related Spectroscopies. J. Phys.: Conf. Ser. 2013, 430, 012007 10.1088/1742-6596/430/1/012007.

[ref49] Adler S. B. Chemical Expansivity of Electrochemical Ceramics. J. Am. Ceram. Soc. 2001, 84, 2117–2119. 10.1111/j.1151-2916.2001.tb00968.x.

[ref50] Moreno R.; García P.; Zapata J.; Roqueta J.; Chaigneau J.; Santiso J. Chemical Strain Kinetics Induced by Oxygen Surface Exchange in Epitaxial Films Explored by Time-Resolved X-Ray Diffraction. Chem. Mater. 2013, 25, 3640–3647. 10.1021/cm401714d.

[ref51] Shannon R. D. Revised Effective Ionic Radii and Systematic Studies of Interatomic Distances in Halides and Chalcogenides. Acta Crystallogr., A 1976, 32, 751–767. 10.1107/S0567739476001551.

[ref52] Gibbs A. S.; Knight K. S.; Lightfoot P. High-Temperature Phase Transitions of Hexagonal YMnO_3_. Phys. Rev. B 2011, 83, 094111 10.1103/PhysRevB.83.094111.

[ref53] Tomczyk M.; Senos A. M. O. R.; Reaney I. M.; Vilarinho P. M. Reduction of Microcracking in YMnO_3_ Ceramics by Ti Substitution. Scr. Mater. 2012, 67, 427–430. 10.1016/j.scriptamat.2012.04.042.

[ref54] Skjærvø S. H.; Meier Q. N.; Feygenson M.; Spaldin N. A.; Billinge S. J. L.; Bozin E. S.; Selbach S. M. Unconventional Continuous Structural Disorder at the Order-Disorder Phase Transition in the Hexagonal Manganites. Phys. Rev. X 2019, 9, 031001 10.1103/PhysRevX.9.031001.

[ref55] Fennie C. J.; Rabe K. M. Ferroelectric Transition in YMnO_3_ from First Principles. Phys. Rev. B: Condens. Matter Mater. Phys. 2005, 72, 100103 10.1103/PhysRevB.72.100103.

[ref56] Lilienblum M.; Lottermoser T.; Manz S.; Selbach S. M.; Cano A.; Fiebig M. Ferroelectricity in the Multiferroic Hexagonal Manganites. Nat. Phys. 2015, 11, 1070–1073. 10.1038/nphys3468.

[ref57] Koettgen J.; Zacherle T.; Grieshammer S.; Martin M. Ab Initio Calculation of the Attempt Frequency of Oxygen Diffusion in Pure and Samarium Doped Ceria. Phys. Chem. Chem. Phys. 2017, 19, 9957–9973. 10.1039/c6cp04802a. 28361150

[ref58] Krauskopf T.; Muy S.; Culver S. P.; Ohno S.; Delaire O.; Shao-Horn Y.; Zeier W. G. Comparing the Descriptors for Investigating the Influence of Lattice Dynamics on Ionic Transport Using the Superionic Conductor Na3PS4-XSex. J. Am. Chem. Soc. 2018, 140, 14464–14473. 10.1021/jacs.8b09340. 30284822

[ref59] Bouwmeester H. J. M.; Kruidhof H.; Burggraaf A. Importance of the Surface Exchange Kinetics as Rate Limiting Step in Oxygen Permeation through Mixed-Conducting Oxides. Solid State Ionics 1994, 72, 185–194. 10.1016/0167-2738(94)90145-7.

[ref60] Fischer E.; Hertz J. L. Measurability of the Diffusion and Surface Exchange Coefficients Using Isotope Exchange with Thin Film and Traditional Samples. Solid State Ionics 2012, 218, 18–24. 10.1016/j.ssi.2012.05.003.

[ref61] Yang Q.; Burye T. E.; Lunt R. R.; Nicholas J. D. In Situ Oxygen Surface Exchange Coefficient Measurements on Lanthanum Strontium Ferrite Thin Films via the Curvature Relaxation Method. Solid State Ionics 2013, 249-250, 123–128. 10.1016/j.ssi.2013.07.025.

[ref62] Chen T.; Harrington G. F.; Masood J.; Sasaki K.; Perry N. H. Emergence of Rapid Oxygen Surface Exchange Kinetics during in Situ Crystallization of Mixed Conducting Thin Film Oxides. ACS Appl. Mater. Interfaces 2019, 11, 9102–9116. 10.1021/acsami.8b21285. 30676719

[ref63] Skiba E. J.; Chen T.; Perry N. H. Simultaneous Electrical, Electrochemical, and Optical Relaxation Measurements of Oxygen Surface Exchange Coefficients: Sr(Ti,Fe)O 3–d Film Crystallization Case Study. ACS Appl. Mater. Interfaces 2020, 12, 48614–48630. 10.1021/acsami.0c14265. 33075221

[ref64] Stangl A.; Riaz A.; Rapenne L.; Caicedo J. M.; De Dios Sirvent J.; Baiutti F.; Jiménez C.; Tarancón A.; Mermoux M.; Burriel M. Tailored Nano-Columnar La2NiO4cathodes for Improved Electrode Performance. J. Mater. Chem. A 2022, 10, 2528–2540. 10.1039/d1ta09110g.

[ref65] Yang G.; Kim S. Y.; Sohn C.; Keum J. K.; Lee D. Influence of Heterointerfaces on the Kinetics of Oxygen Surface Exchange on Epitaxial La1.85Sr0.15CuO4 Thin Films. Appl. Sci. 2021, 11, 3778 10.3390/app11093778.

[ref66] Acosta M.; Baiutti F.; Wang X.; Cavallaro A.; Wu J.; Li W.; Parker S. C.; Aguadero A.; Wang H.; Tarancón A.; MacManus-Driscoll J. L. Surface Chemistry and Porosity Engineering through Etching Reveal Ultrafast Oxygen Reduction Kinetics below 400 °C in B-Site Exposed (La,Sr)(Co,Fe)O3 Thin-Films. J. Power Sources 2022, 523, 230983 10.1016/j.jpowsour.2022.230983.

[ref67] Cichy K.; Świerczek K. Influence of Doping on the Transport Properties of Y_1-x_Ln_x_MnO_3+δ_ (Ln: Pr, Nd). Crystals 2021, 11, 510 10.3390/cryst11050510.

[ref68] Li M.; Pietrowski M. J.; De Souza R. A.; Zhang H.; Reaney I. M.; Cook S. N.; Kilner J. A.; Sinclair D. C. A Family of Oxide Ion Conductors Based on the Ferroelectric Perovskite Na_0.5_Bi_0.5_TiO_3_. Nat. Mater. 2014, 13, 31–35. 10.1038/nmat3782. 24193663

[ref69] Kharton V. V.; Marques F. M. B.; Atkinson A. Transport Properties of Solid Oxide Electrolyte Ceramics: A Brief Review. Solid State Ionics 2004, 174, 135–149. 10.1016/j.ssi.2004.06.015.

