# Supplementary material for: Oxidation Kinetics of Nanocrystalline Hexagonal RMn1–xTixO3 (R = Ho, Dy)
Source: ACS Appl Mater Interfaces. 2023 Aug 28;15(36):42439–48. doi: 10.1021/acsami.3c06020 (PMC10510046; doi:10.1021/acsami.3c06020)
Supplement: Supplementary file 1 — am3c06020_si_001.pdf [file am3c06020_si_001.pdf]

Supporting Information for:

Oxidation kinetics of nanocrystalline hexagonal $R\mathrm{Mn}_{1-x}\mathrm{Ti}_x\mathrm{O}_3$ (R = Ho, Dy)

Frida Hemstad Danmo,[1] Inger-Emma Nylund,[1] Aamund Westermoen, [1] Kenneth P. Marshall,[2] Dragos Stoian,[2] Tor Grande,[1] Julia Glaum,[1] and Sverre M. Selbach[1,]*

[1]Department of Materials Science and Engineering, NTNU Norwegian University of Science and Technology, NO-7491 Trondheim, Norway.

[2]The Swiss-Norwegian Beamlines (SNBL), European Synchrotron Radiation Facility, Grenoble 38043, France

[*]E-mail: selbach@ntnu.no

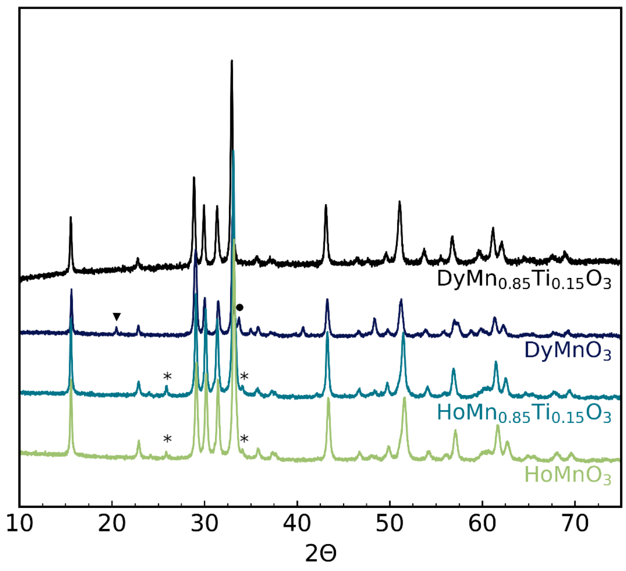

Figure S1: X-ray diffractograms of as-synthesized materials with compositions and labels explained in Table 1. The asterisks (*) indicate reflections originating from the orthorhombic perovskite *Pnma* phase, the triangle (▼) indicate reflections from an unidentified phase, and the circle (●) indicates the presence of Dy_2O_3.

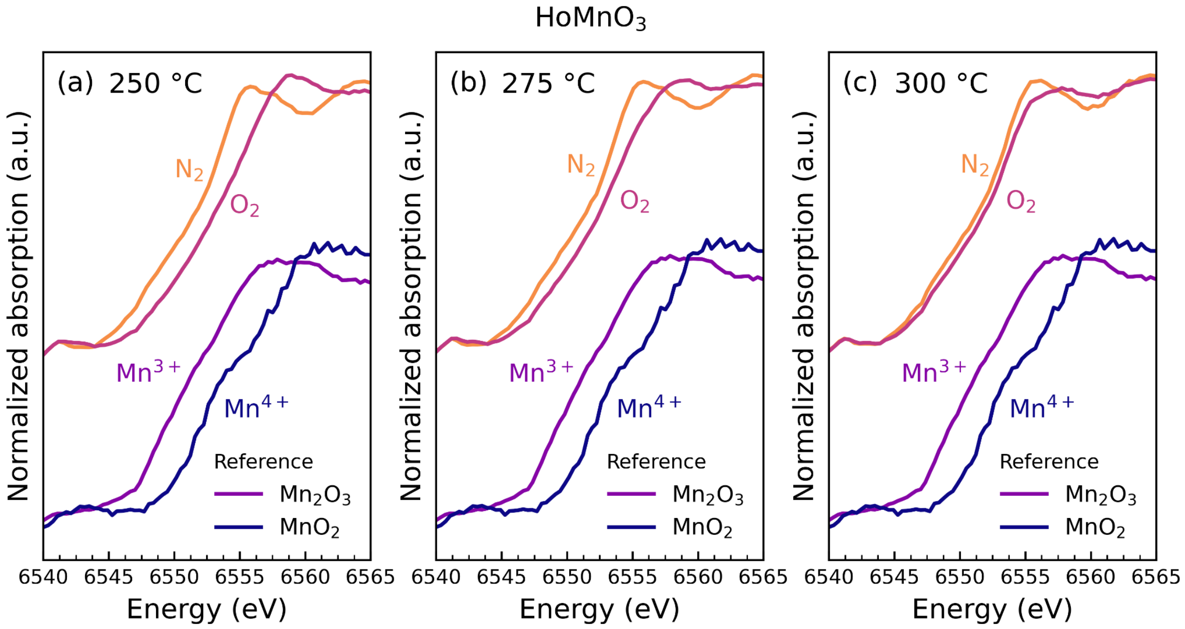

Figure S2: Normalized X-ray absorption near-edge structure (XANES) spectra at the Mn *K*-edge of $HoMnO_3$ before (N_2) and after oxidation (O_2). The spectra of Mn_2O_3 and MnO_2 are shown as references.

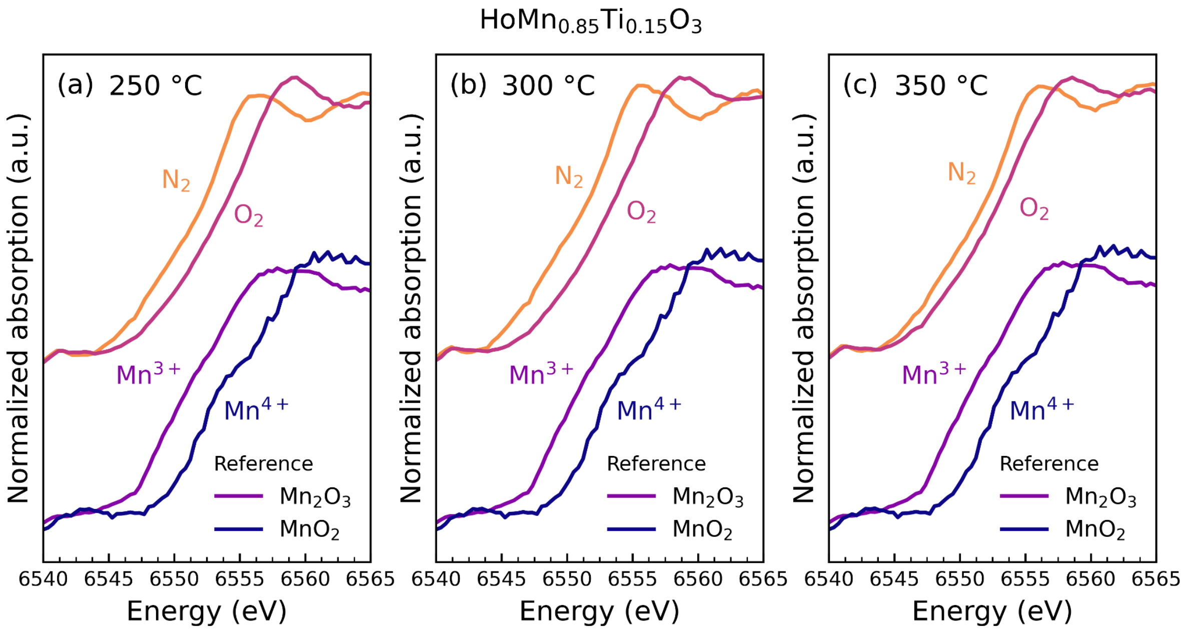

Figure S3: Normalized X-ray absorption near-edge structure (XANES) spectra at the Mn *K*-edge of $HoMn_{0.85}Ti_{0.15}O_3$ before (N_2) and after oxidation (O_2). The spectra of Mn_2O_3 and MnO_2 are shown as references.

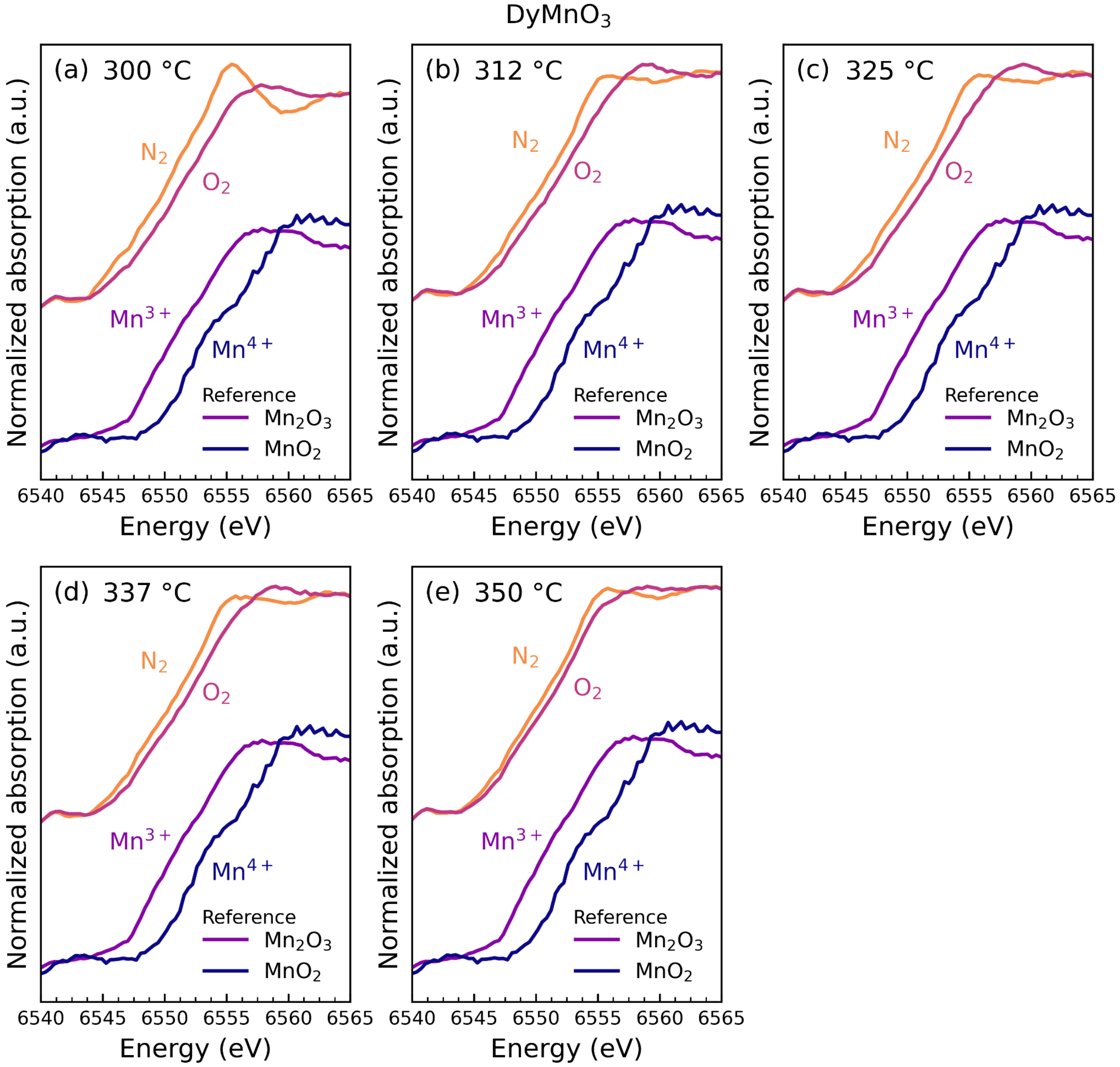

Figure S4: Normalized X-ray absorption near-edge structure (XANES) spectra at the Mn *K*-edge of $DyMnO_3$ before (N_2) and after oxidation (O_2). The spectra of Mn_2O_3 and MnO_2 are shown as references.

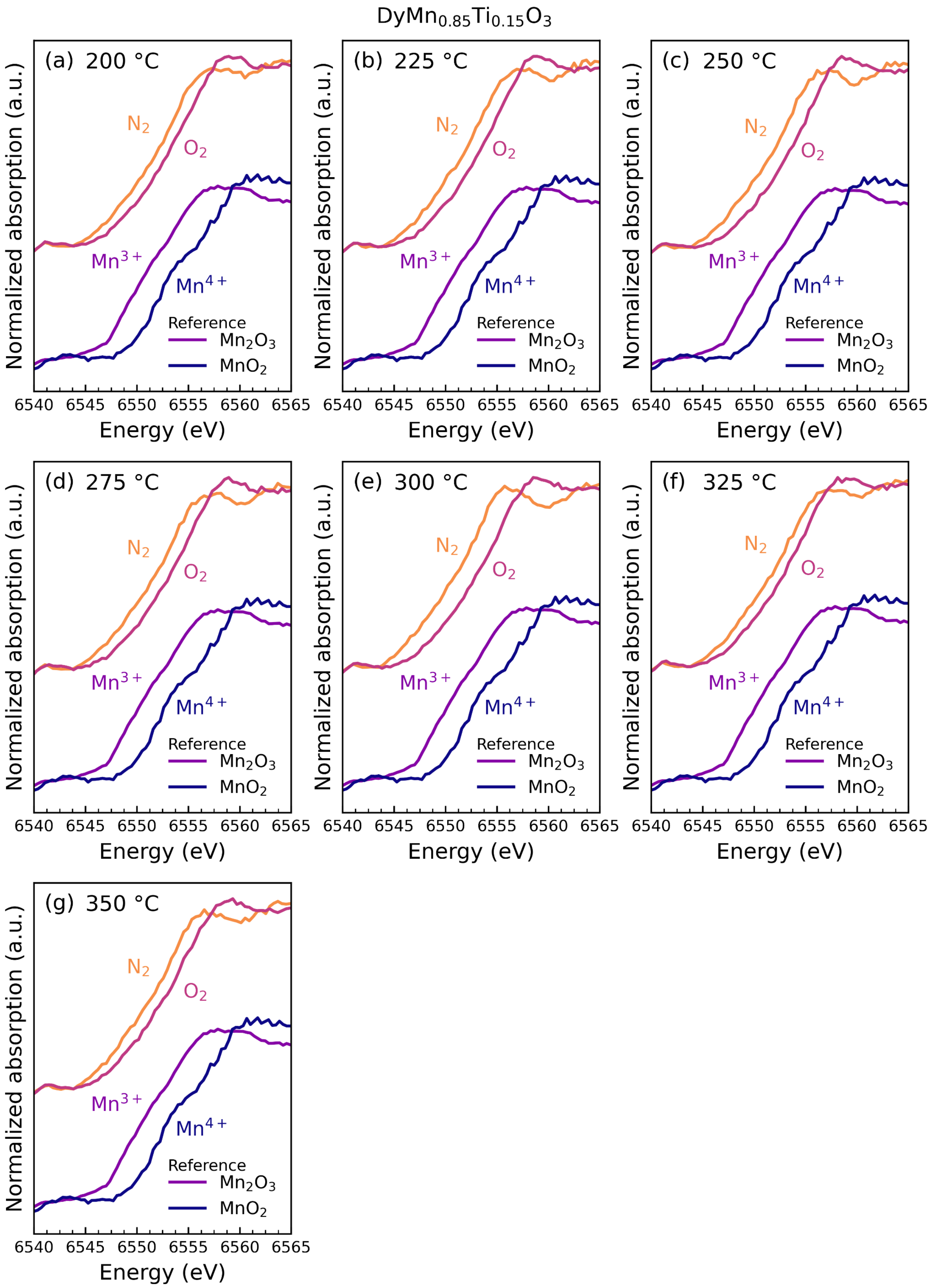
DyMn0.85Ti0.15O3
(a) 200 °C
(b) 225 °C
(c) 250 °C
(d) 275 °C
(e) 300 °C
(f) 325 °C
(g) 350 °C
N2
O2
Mn3+
Mn4+
Reference
Mn2O3
MnO2
Normalized absorption (a.u.)
Energy (eV)
6540 6545 6550 6555 6560 6565

Figure S5: Normalized X-ray absorption near-edge structure (XANES) spectra at the Mn *K*-edge of $DyMn_{0.85}Ti_{0.15}O_3$ before (N_2) and after oxidation (O_2). The spectra of Mn_2O_3 and MnO_2 are shown as references.

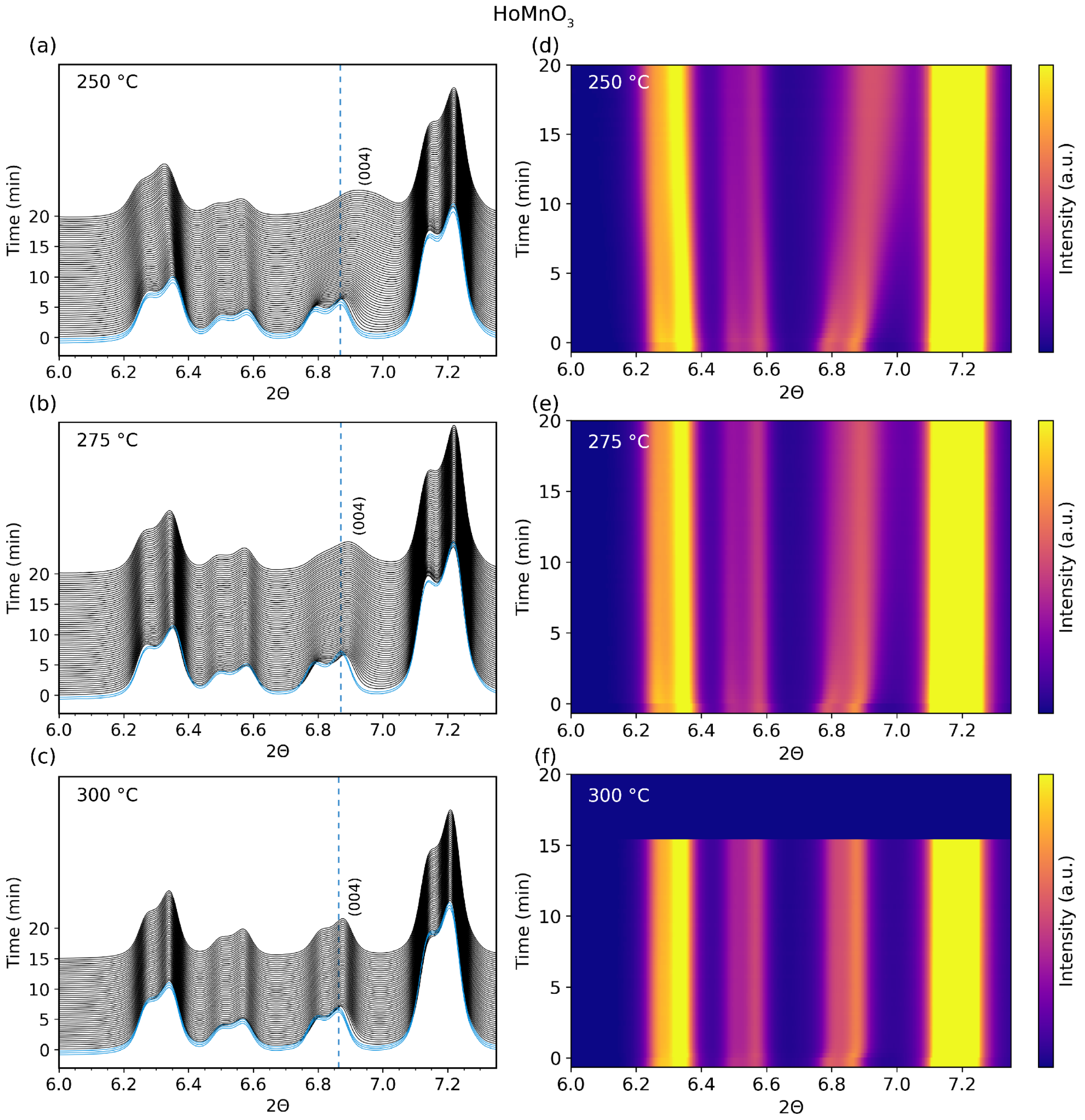

Figure S6: X-ray diffractograms and 2D contour plots of $HoMnO_3$ as a function of time after *in situ* switching of atmosphere from N_2 (blue) to O_2 (black) at different temperatures. The baseline intensity at 6.0° 2θ in (a)-(c) indicate the time in min. after switching from N_2 to O_2 purge gas. Vertical dashed lines (blue) indicate the initial position of the (0 0 4) reflection measured in N_2.

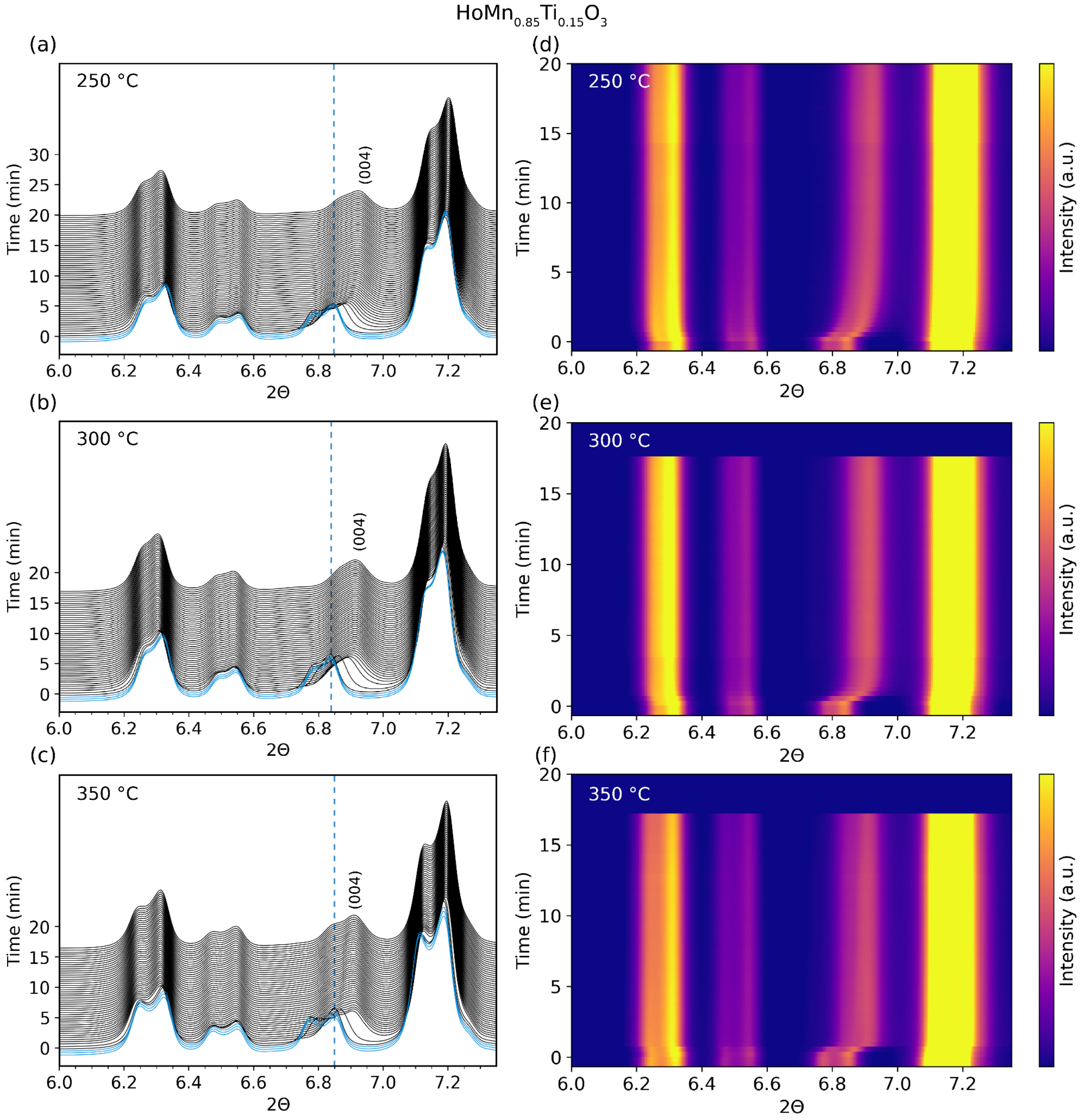

Figure S7: X-ray diffractograms and 2D contour plots of $HoMn_{0.85}Ti_{0.15}O_3$ as a function of time after *in situ* switching of atmosphere from N_2 (blue) to O_2 (black) at different temperatures. The baseline intensity at 6.0° 2θ in (a)-(c) indicate the time in min. after switching from N_2 to O_2 purge gas. Vertical dashed lines (blue) indicate the initial position of the (0 0 4) reflection measured in N_2.

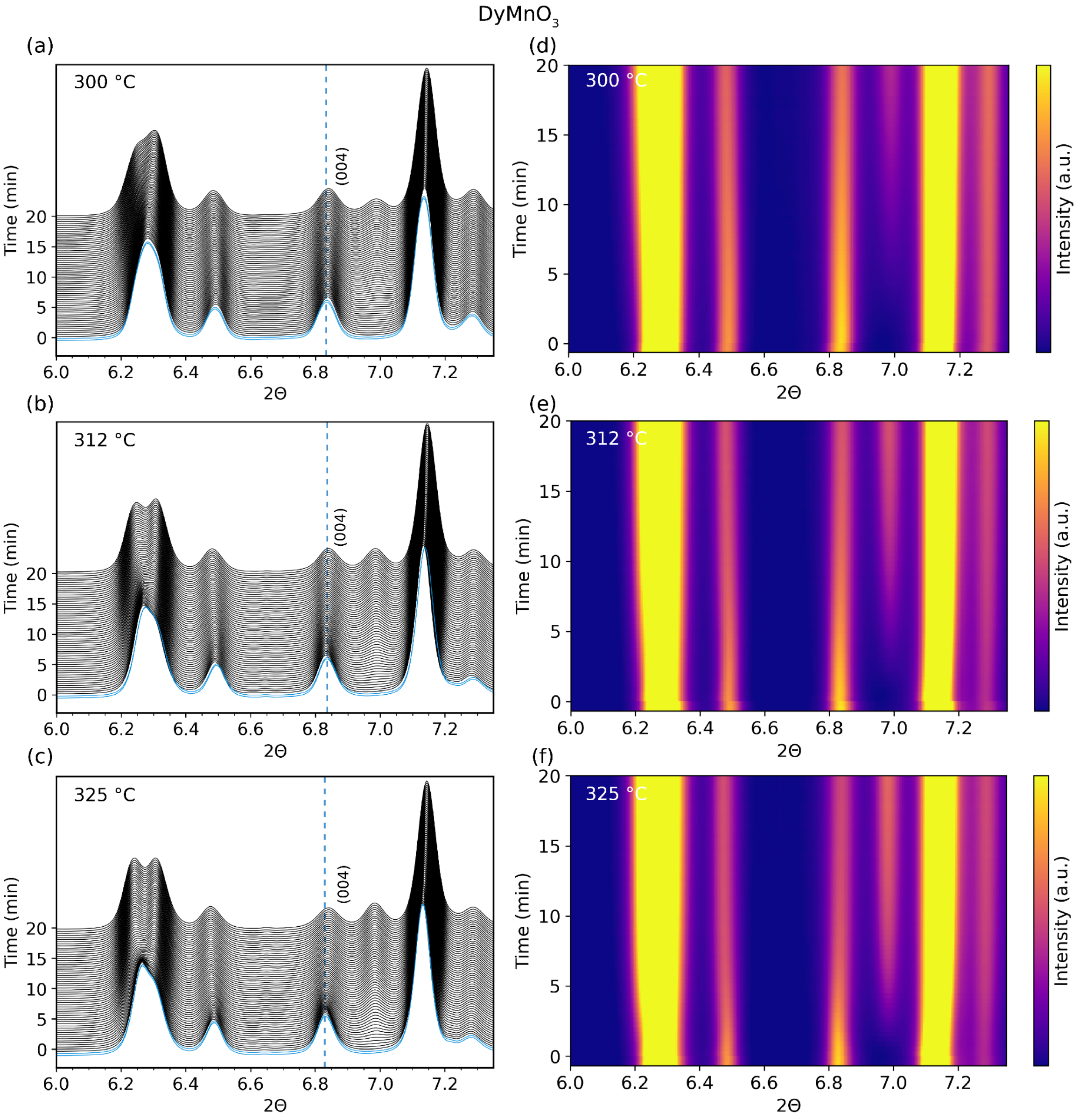

Figure S8: X-ray diffractograms and 2D contour plots of $DyMnO_3$ as a function of time after *in situ* switching of atmosphere from N_2 (blue) to O_2 (black) at different temperatures. The baseline intensity at 6.0° 2θ in (a)-(c) indicate the time in min. after switching from N_2 to O_2 purge gas. Vertical dashed lines (blue) indicate the initial position of the (0 0 4) reflection measured in N_2.

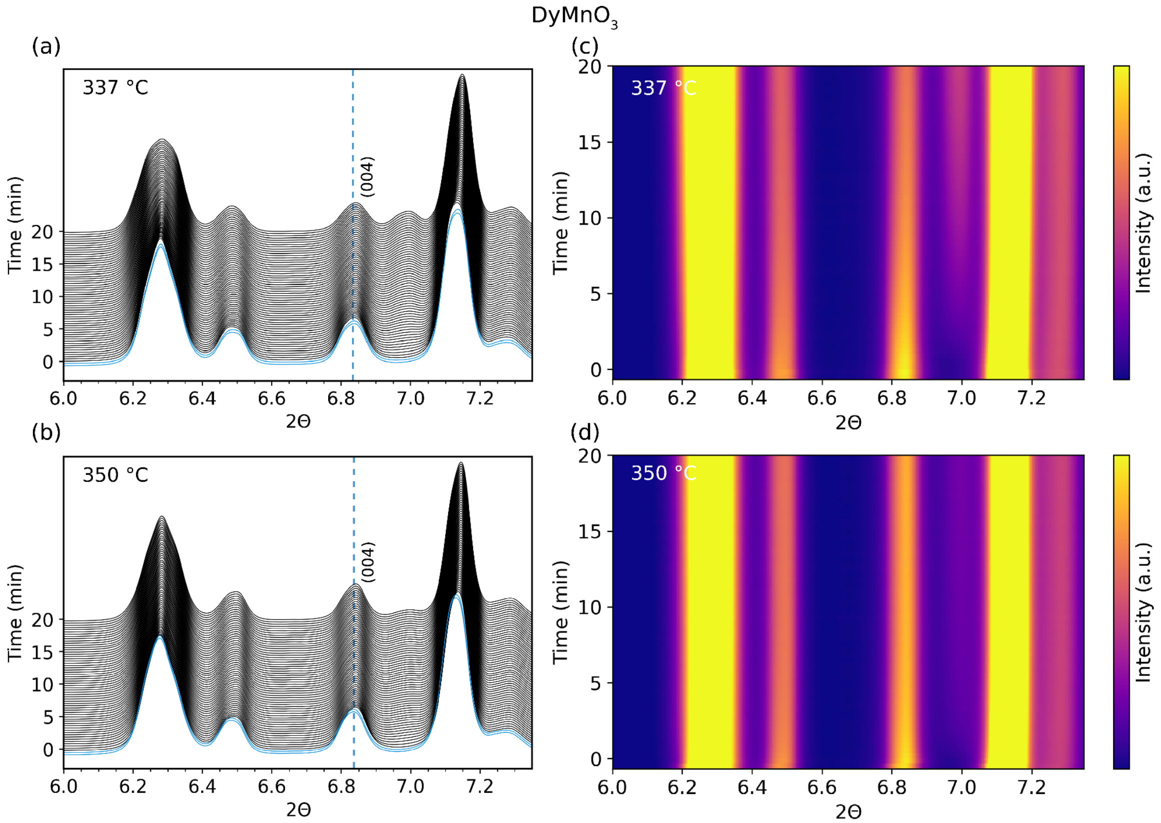

Figure S9: X-ray diffractograms and 2D contour plots of $DyMnO_3$ as a function of time after *in situ* switching of atmosphere from N_2 (blue) to O_2 (black) at different temperatures. The baseline intensity at 6.0° 2θ in (a)-(b) indicate the time in min. after switching from N_2 to O_2 purge gas. Vertical dashed lines (blue) indicate the initial position of the (0 0 4) reflection measured in N_2.

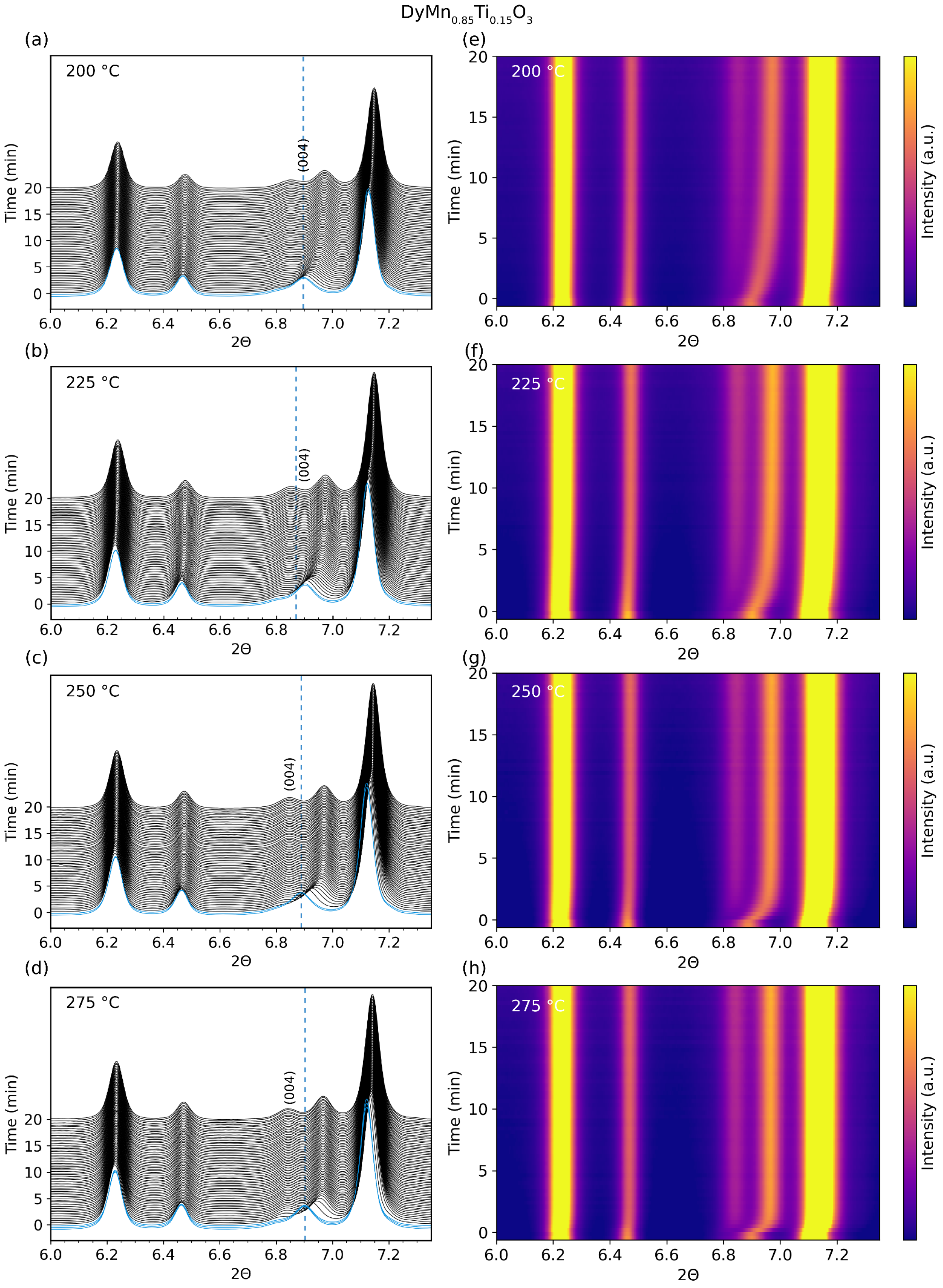

Figure S10: X-ray diffractograms and 2D contour plots of DyMn$_{0.85}$Ti$_{0.15}$O$_3$ as a function of time after *in situ* switching of atmosphere from N_2 (blue) to O_2 (black) at different temperatures. The baseline intensity at 6.0°

2θ in (a)-(d) indicate the time in min. after switching from N_2 to O_2 purge gas. Vertical dashed lines (blue) indicate the initial position of the (0 0 4) reflection measured in N_2.

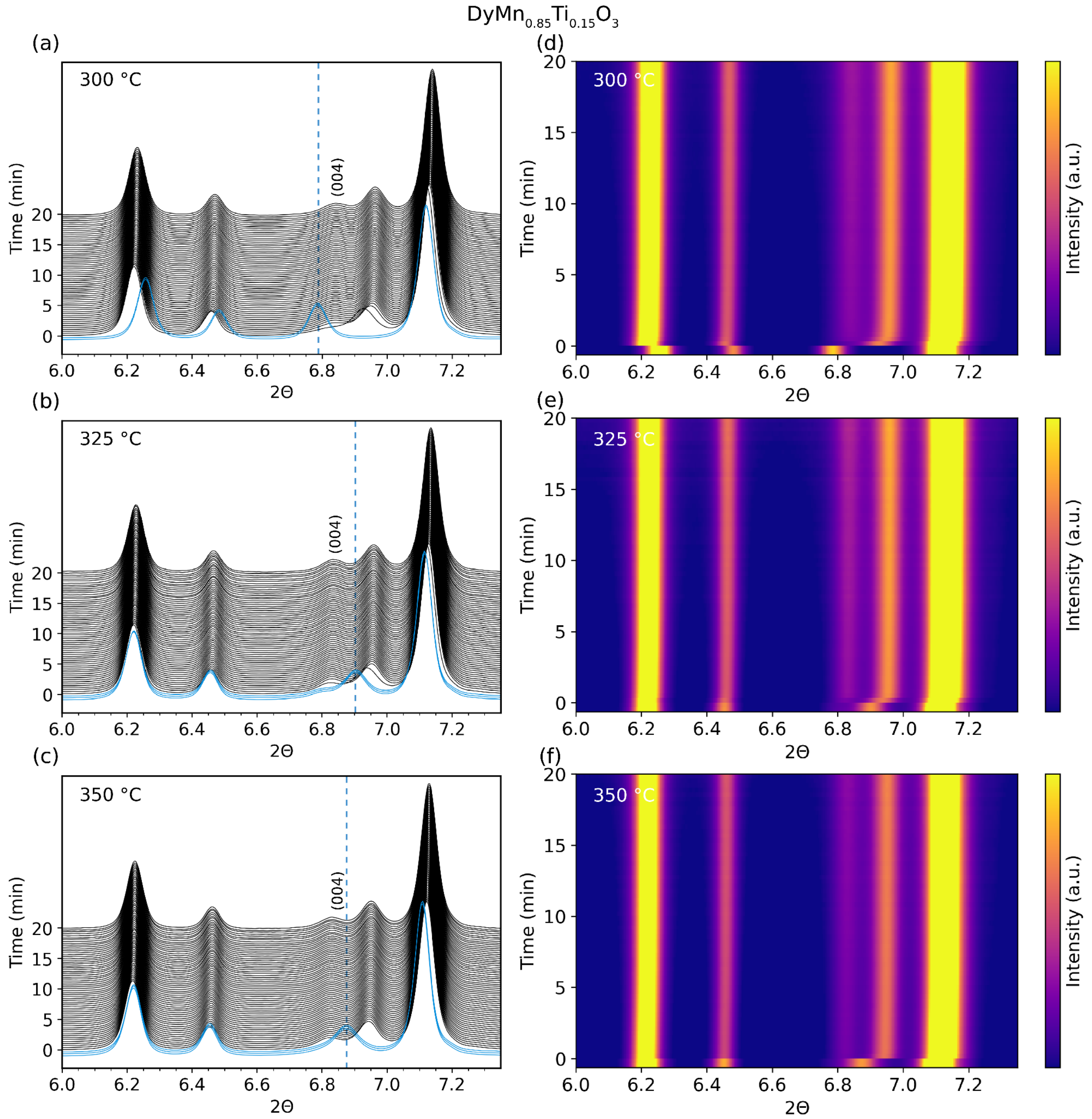

Figure S11: X-ray diffractograms and 2D contour plots of $DyMn_{0.85}Ti_{0.15}O_3$ as a function of time after *in situ* switching of atmosphere from N_2 (blue) to O_2 (black) at different temperatures. The baseline intensity at 6.0° 2θ in (a)-(d) indicate the time in min. after switching from N_2 to O_2 purge gas. Vertical dashed lines (blue) indicate the initial position of the (0 0 4) reflection measured in N_2.

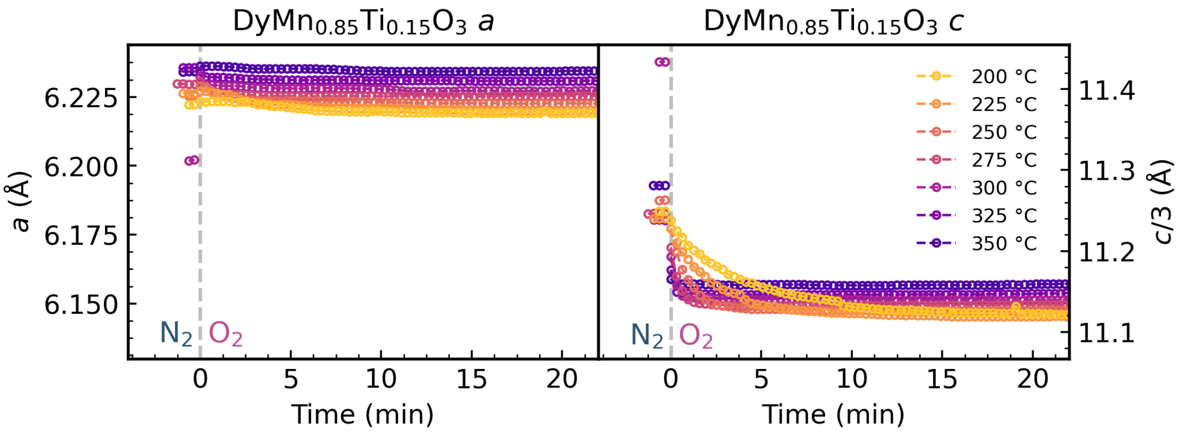

Figure S12: Refined lattice parameters *a* and *c* as a function of time after switching from N_2 to O_2 atmosphere for $DyMn_{0.85}Ti_{0.15}O_3$.
